# Genotyping by sequencing provides new insights into the diversity of Napier grass (*Cenchrus purpureus*) and reveals variation in genome-wide LD patterns between collections

**DOI:** 10.1038/s41598-019-43406-0

**Published:** 2019-05-06

**Authors:** Meki S. Muktar, Abel Teshome, Jean Hanson, Alemayehu T. Negawo, Ermias Habte, Jean-Baka Domelevo Entfellner, Ki-Won Lee, Chris S. Jones

**Affiliations:** 1 Feed and Forage Development, International Livestock Research Institute, Addis Ababa, Ethiopia; 2 Teagasc|CELUP Crop Research, Oak Park, Carlow R93 XE12 Ireland; 3 Grassland and Forages Division, National Institute of Animal Science, Rural Development Administration, Cheonan, 31000 Republic of Korea; 4 Biosciences eastern and central Africa, International Livestock Research Institute, Nairobi, Kenya; 5 Feed and Forage Development, International Livestock Research Institute, Nairobi, Kenya

**Keywords:** Natural variation in plants, Agricultural genetics

## Abstract

Napier grass is an important tropical forage-grass and of growing potential as an energy crop. One-hundred-five Napier grass accessions, encompassing two independent collections, were subjected to genotyping by sequencing which generated a set of high-density genome-wide markers together with short sequence reads. The reads, averaging 54 nucleotides, were mapped to the pearl millet genome and the closest genes and annotation information were used to select candidate genes linked to key forage traits. 980 highly polymorphic SNP markers, distributed across the genome, were used to assess population structure and diversity with seven-subgroups identified. A few representative accessions were selected with the objective of distributing subsets of a manageable size for further evaluation. Genome-wide linkage disequilibrium (LD) analyses revealed a fast LD-decay, on average 2.54 kbp, in the combined population with a slower LD-decay in the ILRI collection compared with the EMBRAPA collection, the significance of which is discussed. This initiative generated high-density markers with a good distribution across the genome. The diversity analysis revealed the existence of a substantial amount of variation in the ILRI collection and identified some unique materials from the EMBRAPA collection, demonstrating the potential of the overall population for further genetic and marker-trait-association studies.

## Introduction

Napier grass (*Cenchrus purpureus* (Schumach.) Morrone syn. *Pennisetum purpureum* Schumach.), also called elephant grass, is one of the most important tropical forage grasses suited for zero grazing and mainly used as a cut-and-carry feed. It is native to Sub-Saharan Africa and widely distributed across the global tropics, mainly as a forage species^1–4^ and also as a potential energy crop^3,5,6^. Napier grass is known for its good attributes including high biomass productivity and quality^7,8^, year-round availability under irrigated conditions^9^, resistance to most pests and diseases^10,11^, ease of establishment and rapid propagation^8^ and fast regrowth capacity^12^.

Napier grass is a C_4_ species which thrives in open, arid and marginal lands, environments that are becoming more prevalent as a consequence of climate change^13,14^. Consequently, it is one of the key forages for small-scale farmers, in most of the Eastern, Central and Southern African countries^8^. However, the performance of current varieties is being significantly negatively impacted, principally as a result of erratic weather conditions arising from changes in climatic conditions^15,16^. Furthermore, biotic stresses such as Napier grass stunt and head smut diseases are rapidly spreading and causing significant yield losses, especially in Central and Eastern African countries^2,17^. Consequently, there is a pressing need to develop new varieties which are capable of withstanding the current and future environmental challenges and are resilient in the face of major diseases. Napier grass is yet to be fully domesticated and consequently lags behind other grasses in terms of genetic and genomic tools^18^. To date, Napier grass breeding initiatives have relied heavily on field evaluations, which has made breeding efforts relatively slow and arduous due to the architecture and perennial nature of the species^8,19^. In addition, the outcrossing nature, self-incompatibility^20^ and higher ploidy level (2n = 4x = 28) of Napier grass have further inhibited conventional breeding approaches in this species^21^. However, the application of advanced genomic tools and “speed breeding” techniques offers the opportunity to fast-forward the breeding cycles and open up the avenue to fully exploit this species as an alternative forage and energy crop.

Up until the last decade, developing genomic tools was an expensive and time-consuming endeavour, hence only a few grass species such as maize, wheat and rice benefited. To date Napier grass has only had a handful of random molecular markers applied, mainly targeting the assessment of genetic diversity^22–26^. Fortunately, recent advances in next generation sequencing have allowed the application of genotyping by sequencing (GBS) approaches in orphan crops, such as Napier grass, which have limited genomic information. GBS produces a large amount of high-quality genome-wide genetic markers which are suitable for diversity analysis, marker-trait associations^27,28^ and genomic prediction^29^ and have been used in accelerating genetic gain in crop breeding projects^27,30^. The GBS approach, which enabled the identification of high quality genome-wide simple sequence repeat (SSR) and single nucleotide polymorphism (SNP) markers, has recently been applied to Napier grass^25,31^ leading to the construction of the first high density linkage map in this species^31^.

In the present study, we report on the development of genome-wide and sequence-based molecular markers for 105 Napier grass accessions held in the International Livestock Research Institute (ILRI) genebank using the DArTseq platform. The DArTseq sequencing technology uses a combination of genome complexity reduction, employing restriction enzymes, together with next generation sequencing (NGS) and produces high-density genome-wide dominant (SilicoDArT) and co-dominant (SNP) markers^32,33^. The generation of these markers together with the recently reported reference genome of pearl millet (*Pennisetum glaucum*)^34^ enabled us to undertake an analysis of genetic diversity, linkage disequilibrium (LD) and LD-decay, and to develop a representative core and subsets of genotypes, for both optimal-water and water-deficit conditions, from the Napier grass collection. We also show how we have exploited the reference genome of pearl millet together with genomic information of foxtail millet (*Setaria italica*)^35^, which are the two species with genome sequence information that are most closely related to Napier grass^35^, to determine the chromosomal location of thousands of DArTseq markers, identify the closest gene with which the markers align and putatively annotate the marker.

## Results

### Napier grass population

A combined Napier grass population from the International Livestock Research Institute (ILRI) forage genebank and the Brazilian Agricultural Research Corporation (EMBRAPA) was used in the study. The collection from the ILRI forage genebank represents a diverse set of genotypes assembled from a range of environments and origins (Supplementary Table S1) which is maintained *in situ* at the Bishoftu and Ziway (Batu) sites in Ethiopia. The collection incorporates a high amount of genetic diversity^24^ and is very variable in terms of agronomic and morphological traits^19^. The collection also incorporates eight *P. purpureum* × *P. glaucum* hybrids (Table 1).

**Table 1:** Napier grass collections used in the study.

ILRI collections		EMBRAPA collections	
*Pennisetum purpureum*	Hybrid (*P. purpureum* × *P. glaucum*)	Elite lines	Accessions
52	8	25	20

The detail of each accession is shown in the Supplementary Table S1.

The collection acquired from EMBRAPA also represents a unique set of Napier grass accessions collected from seven different countries in Central and South America^4,24^ and includes 25 elite lines from the EMBRAPA active breeding program (Table 1). This collection has been characterized for agronomic, morphological and molecular traits^36–38^ and was introduced into the ILRI genebank after being analyzed for their distinctiveness using SSR markers^24^.

### Genotyping napier grass accessions by GBS

A total of 116,190 SilicoDArT markers were called on the 105 Napier grass accessions, with an average call rate of 95%. The expected heterozygosity (He) ranged from 0 to 0.5 while the polymorphic information content (PIC) ranged from 0 to 0.38, where 0.5 is the maximum He and PIC value for biallelic markers. The average He and PIC values were 0.24 and 0.19, respectively. Missing values ranged from 1 to 17% for accessions, while they ranged from 0 to 30% for the SilicoDArT markers. The length of the short sequence reads corresponding with each SilicoDArT marker ranged from 20 to 69 nucleotides (nt), with an average of 52 nt.

A total of 85,452 SNP markers were called on the accessions with an average call rate of 87%. The He values ranged from 0 to 0.5 with an average of 0.13 and PIC values ranged from 0 to 0.38 with an average of 0.11. Missing values ranged from 0 to 59% with an average of 15% for SNP markers, and from 6 to 74% with an average of 15% for accessions. Accession ILRI_16621 had the highest missing value content (74%) and was excluded from further analysis. The length of the short sequence reads corresponding to the SNP markers ranged from 20 to 69 nt, with an average of 58 nt.

**Figure 1:**
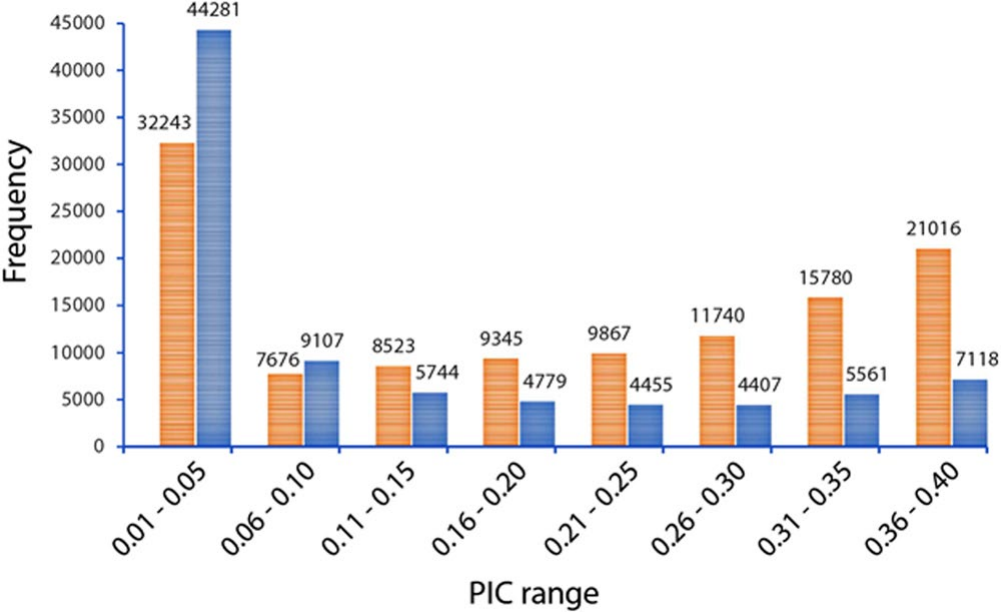
Distribution of polymorphic information content (PIC) values for the SilicoDArT (orange) and SNP (blue) markers.

Approximately 42% (48,536) of the SilicoDArT and 20% (17,086) of the SNP markers had a PIC value above 0.25. The number of SilicoDArT markers within the PIC range of [0.26, 0.30], [0.31, 0.35], and [0.36, 0.40] were more than double, almost triple, when compared to the SNP markers (Fig. 1).

### Putative physical map position of markers

The genome-wide short sequences corresponding to the SilicoDArT and SNP markers were mapped to the genome of pearl millet as there is no genome sequence information available for Napier grass. Pearl millet and foxtail millet are the two species with genome sequence information that are most closely related to Napier grass^34,39^.

**Figure 2:**
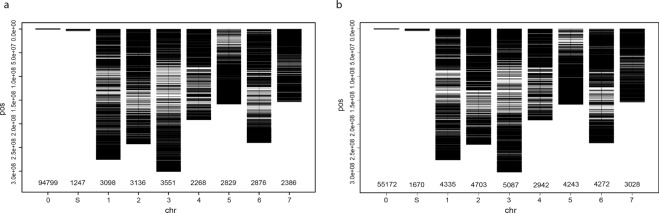
Genome-wide distribution of SilicoDArT (**a**) and SNP (**b**) markers across the seven chromosomes of the pearl millet genome. The markers that were not mapped are indicated by a 0, and those markers that were mapped onto different scaffolds are indicated by an S. The number of markers mapped per chromosome is shown on the x-axis.

The number of mapped markers per chromosome ranged from 2,268 to 3,551 for the SilicoDArT markers, and from 2,942 to 5,087 for the SNP markers. In both marker sets, the highest number of markers mapped onto chromosome 3 while the lowest mapped to chromosome 4, which could be indicative of the relative size of the chromosomes. In general, only 17% (20,144 out of 116,190) of the SilicoDArT markers and 33% (28,610 out of 85,452) of the SNP markers were mapped onto the seven chromosomes of the pearl millet genome. Very few markers, 1,670 SNP markers and 1,247 SilicoDArT markers, were mapped onto different scaffolds (Fig. 2).

Out of the 20,144 SilicoDArT markers with map position information, He values ranged from 0 to 0.5 with an average value of 0.26 while PIC values ranged from 0 to 0.38 with an average value of 0.21. For the 28,610 mapped SNP markers, He and PIC values ranged from 0 to 0.5 and 0 to 0.38 with an average value of 0.22 and 0.18, respectively. More than 45% of the SilicoDArT markers and 25% of the SNP markers had a PIC value above 0.25.

### SNP annotation and candidate gene selection

To annotate the DArT SNP markers, a reciprocal Translated Basic Local Alignment Search Tool (blastx) analysis was run and the results used to draw up a matching table between the proteomes of the two-reference species (pearl millet and foxtail millet). Each transcribed genome contained roughly 40,000 amino acid sequences and most of them had significant hits (i.e. with a BLASTP Expect value of less than 10) in the respective reference species. The reciprocal blastx analysis resulted in the generation of a total of 18,996 homeologous (A, B) pairs where the best hit for protein A in pearl millet is protein B in foxtail millet, and the best hit for B in foxtail millet is the original protein A in pearl millet. The annotations were obtained for foxtail millet from UniProt (free-text gene function and Gene Ontology annotations) and merged with the association list. The association list was correlated with the DArT SNP marker data based on the genomic locations, extracted from the pearl millet reference genome as described above, resulting in 2,256 annotated DArT SNP markers (Supplementary Table S2).

The correlated SNP markers were compared with genes associated with different traits in pearl millet as reported by Varshney *et al*.^34^, which resulted in the identification of 22 genes that are reported to be strongly associated with agronomic traits (*P* values < 10^−10^) and the genetic variance explained ranged from 12 to 24% (Table 2). The identified biomass related genes, including candidates for fresh stover yield, plant population density and plant height, offer good candidates for further testing their association with increased forage production of Napier grass under optimal and water-deficit conditions.

**Table 2:** SilicoDArT and SNP markers correlated with genes associated with different traits in pearl millet (Varshney *et al*.^34^).

SNP_ID	Chr	Pos	Ref	Alt	Closest_gene_PM	Closest_gene_function	Traits	Treatment	P value	R^2^ (%)
9999783	5	65917816	A	G	Pgl_GLEAN_10010048	AP2/ERF domain	1000-Grain Mass (g)	Early stress	2.46E-11	19
8171327	3	3033078	G	T	Pgl_GLEAN_10005840	Ionotropic glutamate receptor	Fresh Stover Yield (t/ha)	Early stress	5.80E-12	12
23610697	6	184529223	C	G	Pgl_GLEAN_10022294	NA	Fresh Stover Yield (t/ha)	Early stress	4.93E-11	12
23598607	5	66047648	T	C	Pgl_GLEAN_10002412	Protein kinase, catalytic domain	Grain Number /m2 (No.)	Control	2.41E-12	20
23588862	5	66273007	T	C	Pgl_GLEAN_10007383	UbiA prenyltransferase family	Grain Number /m2 (No.)	Late stress	9.71E-12	17
9972446	5	60443354	G	A	Pgl_GLEAN_10009273	Fatty acid hydroxylase	Grain Number /m2 (No.)	Early stress	5.39E-11	16
9968140	5	59276838	C	T	Pgl_GLEAN_10025573	Alcohol dehydrogenase superfamily, zinc-type	Grain Number /m2 (No.)	Early stress	1.62E-11	17
23640298	3	5347418	T	G	Pgl_GLEAN_10000839	Peptidase S8/S53, subtilisin/kexin/sedolisin	Grain Number /Panicle (No.)	Control	6.91E-11	15
23588558	5	64510034	C	T	Pgl_GLEAN_10002983	Phospholipase D/Transphosphatidylase	Grain Number /Panicle (No.)	Control	3.09E-11	18
23602204	5	67552785	G	A	Pgl_GLEAN_10006368	Protein kinase, catalytic domain	Panicle Number (‘000/ha)	Control	1.63E-11	18
23623063	3	8675580	T	A	Pgl_GLEAN_10008425	Proteasome, alpha-subunit, N-terminal domain	Panicle Number (‘000/ha)	Control	3.48E-14	24
9967966	2	135590394	A	T	Pgl_GLEAN_10018209	Sodium/solute symporter	Panicle Number (‘000/ha)	Control	8.84E-12	18
23617275	2	72418790	G	C	Pgl_GLEAN_10021161	NA	Panicle Number (‘000/ha)	Early stress	2.99E-11	15
9966416	2	43404632	G	C	Pgl_GLEAN_10021658	NA	Panicle Number (‘000/ha)	Control	4.92E-11	16
23634420	2	2182294	C	T	Pgl_GLEAN_10023314	Zinc finger, RING-type	Panicle Number (‘000/ha)	Control	6.18E-11	16
23615392	4	162832391	G	A	Pgl_GLEAN_10008211	Raffinose synthase	Plant Height (cm)	Control	2.79E-11	17
23618303	4	119817514	G	C	Pgl_GLEAN_10012722	Heat shock protein DnaJ, N-terminal	Plant Height (cm)	Control	6.63E-11	15
9975905	4	78593371	A	G	Pgl_GLEAN_10019616	NA	Plant Height (cm)	Late stress	1.00E-11	12
23624988	4	140312557	C	A	Pgl_GLEAN_10031827	Protein of unknown function DUF914, eukaryotic	Plant Height (cm)	Late stress	1.75E-12	12
23588605	4	34598785	C	T	Pgl_GLEAN_10036604	Ubiquitin-associated/translation elongation factor	Plant Height (cm)	Control	8.23E-13	18
23603442	2	214449399	A	G	Pgl_GLEAN_10031324	NA	Plant Population (‘000/ha)	Late stress	5.38E-13	23
17974203	5	80840668	G	A	Pgl_GLEAN_10038503	Transcription factor, SBP-box	Plant Population (‘000/ha)	Late stress	2.13E-13	23

Chr = chromosome; Pos = position within chromosome; Ref = reference allele; Alt = alternative allele; PM = pearl millet; NA = information not available.

### Estimated linkage disequilibrium across the Napier grass genome

**Figure 3:**
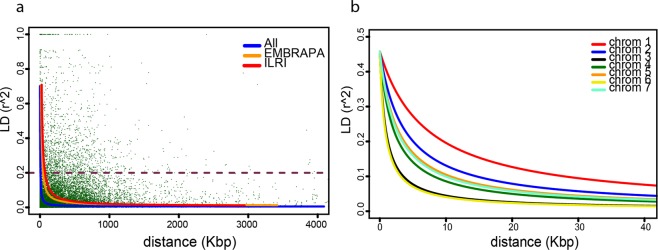
Estimated linkage disequilibrium decay (LD-decay) in 104 Napier grass accessions (blue), 45 EMBRAPA accessions (orange) and 59 ILRI accessions (red) (**a**). In (**b**), the LD-decay per chromosome is shown.

Linkage disequilibrium (LD) was analyzed between pairs of SilicoDArT markers from the same chromosome and then combined to estimate the average LD decay across the A′ genome. The number of markers used in the LD analysis ranged from 1,399 on chromosome 7 to 2,040 on chromosome 3, resulting in a total of 11,720 genome-wide SilicoDArT markers being used. The minor allele frequency (MAF) of the markers was greater than 5% and the missing values were less than 10%. There was a rapid reduction in the magnitude of *r*^2^ as physical map distance between the SilicoDArT markers increased (Fig. 3). In the combined population, the value of *r*^2^ decreased to 0.2 at about 2.54 kbp. In the EMBRAPA (45 accessions) and ILRI (59 accessions) collections, the value of *r*^2^ decreased to 0.2 at about 10.24 kbp and 15.69 kbp, respectively. This indicates the presence of long haplotype blocks in the ILRI collection, which may be due to a fewer number of new meiosis compared to the EMBRAPA collection. Further LD analysis within the EMBRAPA collection revealed a substantial variation in LD and LD-decay between the EMBRAPA genebank collection (20 accessions) and the EMBRAPA elite lines (25 accessions) (Table 1). The average LD-decay across the genome in the EMBRAPA collection was 68.03 kbp, while the LD-decay in the EMBRAPA elite lines was 16.56 kbp (Supplementary Fig. S1).

In the combined population, the LD decays rapidly in chromosome 6 and 3 while it is slower in chromosome 1, suggesting that a larger number of markers are required from chromosome 6 and 3 than from chromosome 1 for genome-wide association studies (GWAS) in Napier grass.

### Diversity in the napier grass populations

To evaluate diversity and population structure, 980 highly polymorphic and independent SNP markers (pruned for LD at *r*^2^ = 0.5) distributed across the genome were selected from the 85,452 genome-wide SNP markers (Supplementary Table S3). The He and PIC values of the markers ranged from 0.23 to 0.50 and 0.21 to 0.38, respectively. The MAF was above 13% and the missing values ranged from 0 to 9% (Supplementary Fig. S2a and c).

**Figure 4:**
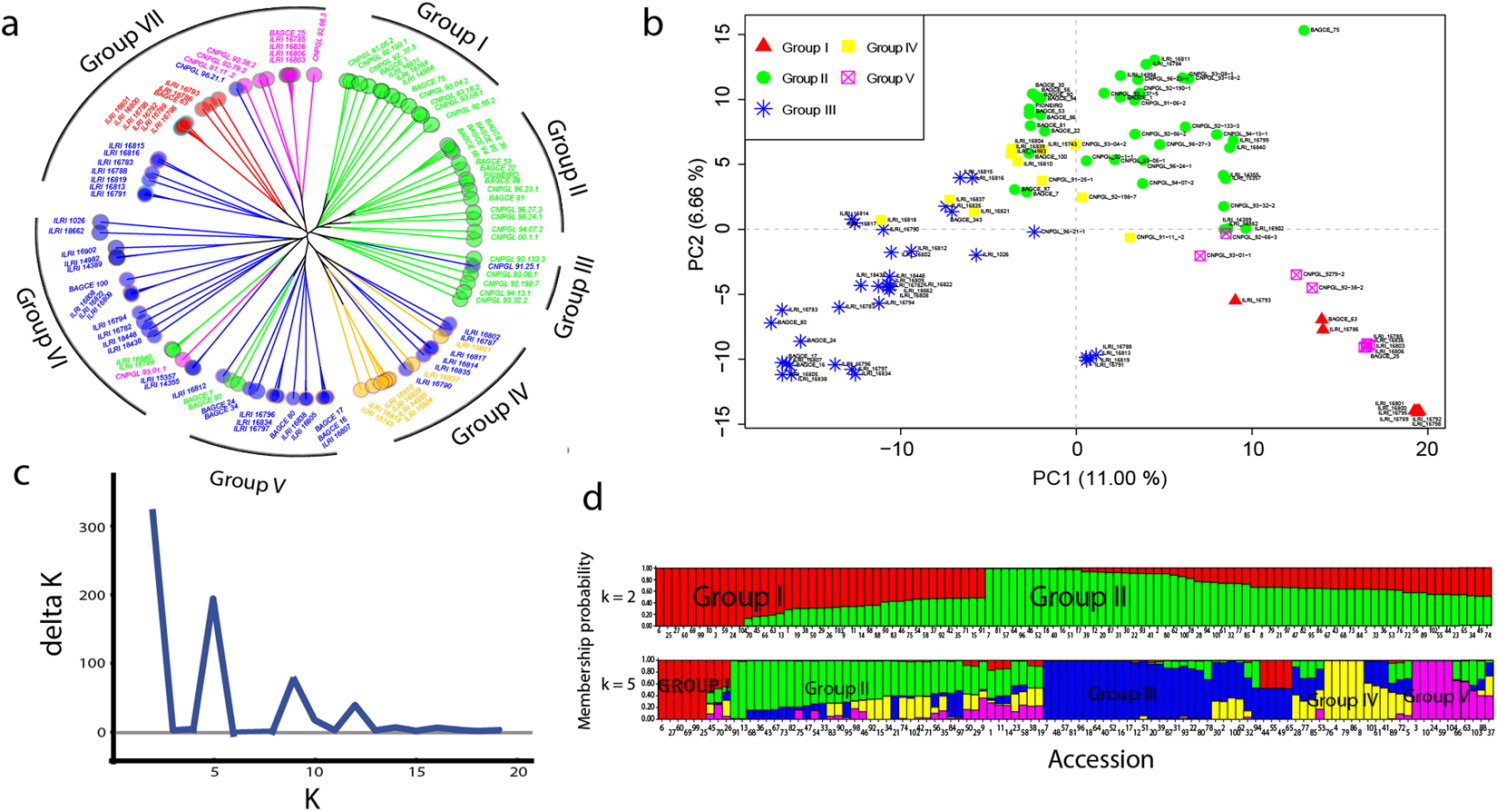
Clusters of the 104 Napier grass accessions using 980 selected SNP markers. (**a**) UPGMA tree showing seven groups; (**b**) PCA plot for PC1 and PC2; (**c**) The delta K suggesting two major groups and up to 5 subgroups; (**d**) Bar plots based on the admixture model in STRUCTURE, for K = 2 and K = 5. The colors in (**a**) and (**b**) are according to the STRUCTURE analysis with k = 5.

The presence of subpopulations within the 104 Napier grass accessions was analyzed, using the 980 SNP markers described above, in the software STRUCTURE. The delta K showed the highest peak at K = 2 (Fig. 4c) indicating the presence of two major groups, with the collection from ILRI predominantly represented in Group I and most of the EMBRAPA collections assigned to Group II. However, there was a second major peak at k = 5 indicating the presence of 5 possible subgroups. At a membership probability threshold of 0.50 considering k = 5, 7 accessions were assigned to Group I, 29 accessions to Group II, 31 accessions to Group III, 8 accessions to Group IV, 7 accessions to Group V and 22 accessions remained admixed (Fig. 4d).

Clustering of the accessions was performed using three different methods and each method was tested for the fidelity of its representation of the original distance matrix between accessions. This was done by plotting the original distance matrix versus the patristic distances between taxa obtained after tree inference. Both Neighbor-Joining (NJ) and Unweighted Pair Group Method with Arithmetic Mean (UPGMA) trees presented a better representation of the distance matrix than hierarchical clustering. UPGMA clustered the accessions into seven sub groups (Fig. 4a), and Groups I, II, III, V, and VI were highly consistent with the STRUCTURE classification (Fig. 4d). Group IV and VI mainly consist of materials from ILRI and Groups I, II and III are mainly EMBRAPA materials, with Groups V and VII containing material from both collections. Six of the EMBRAPA elite lines clustered together in Group III, the remaining elite lines were distributed across Groups I, II and VII. The eight *P. purpureum* × *P. glaucum* hybrids were distributed across groups IV (ILRI_16835 and ILRI_16837), V (ILRI_16834 and ILRI_16838), and VI (ILRI_15357, ILRI_16840, ILRI_18662 and ILRI_14982).

Based on the pedigree information provided by Harris *et al*.^40^, out of the seven accessions which clustered together in Group VII (blue coloured sub group, Fig. 4a), three accessions (ILRI_16815, ILRI_16816 and ILRI_16819) are known to be derived from Merkeron, which is a Napier grass cultivar, derived from an intraspecific cross between a high yielding clone and a dwarf leafy clone, with improved yield and disease resistance^40,41^. Similarly, in Group IV three accessions (ILRI_14983, ILRI_16818 and ILRI_15743) out of six (yellow coloured sub group) are known to be derived from Merkeron.

Out of the seven EMBRAPA (CNPGL) elite lines which clustered together in Group I, four share BAGCE 57 as a common parent, three share BAGCE 49 as a common parent, four share BAGCE 58 as a common parent and another two share BAGCE 37 as a common parent. In Group II, four out of the six CNPGL elite lines share a common parent (BAGCE 5) and out of the four, three share an additional common parent (BAGCE 3). In Group III, all six share a common parent (BAGCE 57), in addition five of them share BAGCE 58 as a common parent and two share another common parent (BAGCE 38) (Supplementary Table S5).

The result from the principal component analysis (PCA) was generally consistent with the above two methods, except that four accessions (ILRI_16788, ILRI_16813, ILRI_16819, and ILRI_16791) from Group III split out and formed a different group. The first and second principal components explained about 18% of the molecular variance (Fig. 4b).

To assess the suitability of SilicoDArT markers for diversity analysis, 1,000 markers (Supplementary Table S4) were selected from the total of 116,190, following a similar procedure to the SNP marker selection. The distribution across the genome and the PIC and He values of these SilicoDArT markers are shown in Supplementary Fig. S2(b and d). Clustering of the 104 accessions using UPGMA revealed seven sub groups, which were very similar to the clusters identified using the 980 SNP markers. Correlation analysis using cophenetic correlation and the dendextend R-package^42^ gave a correlation coefficient of 0.86 (Supplementary Fig. S4) between the dendrograms produced by hierarchical clustering using the 980 SNP markers and 1,000 SilicoDArT markers, indicating that the SilicoDArT markers are comparable to the SNP markers in terms of their discriminating power for diversity analysis.

Analysis of molecular variance (AMOVA) was employed to determine the extent of population differentiation among different groups detected by the different population structure analyses. In most cases the genetic variance among groups was highly significant (*P* < 0.01) while variation among genotypes within each group was not significant. However, the two groups (k = 2) detected by the STRUCTURE analysis showed significant variation both between groups as well as among genotypes within a group. The percentage of variation among groups ranged from 7.60% in the two groups detected by the STRUCTURE analysis to 14.42% in the seven groups detected by UPGMA using the SilicoDArT markers (Table 3).

**Table 3:** Results of the analysis of molecular variance (AMOVA) for groups detected by different population structure analyses.

Methods/markers used in population structure analysis	Source of variation	Degrees of freedom (df)	Sum of squares	Mean sum of squares	Percentage of variation	*P-value*
Two sub groups by STRUCTURE	VBG	1	1958.79	1958.79	7.60	0.001
	VBGWG	102	23258.73	228.03	7.09	0.003
	VWG	104	20336.38	195.54	85.31	0.001
	TV	207	45553.91	220.07	100	
Five subgroups by STRUCTURE	VBG	4	5563.54	1390.88	13.95	0.001
	VBGWG	99	19653.99	198.53	0.65	0.434
	VWG	104	20336.38	195.54	85.40	0.001
	TV	207	45553.91	220.07	100	
Seven subgroups using selected SNPs, and UPGMA tree inference	VBG	6	6360.07	1060.01	13.28	0.001
	VBGWG	97	18857.45	194.41	−0.25	0.518
	VWG	104	20336.38	195.54	86.98	0.001
	TV	207	45553.91	220.07	100	
Seven subgroups using selected SilicoDArTs, and UPGMA tree inference	VBG	6	6765.94	1127.66	14.42	0.001
	VBGWG	97	18451.59	190.22	−1.18	0.669
	VWG	104	20336.38	195.54	86.65	0.001
	TV	207	45553.91	220.07	100	

VBG = Variation between groups; VBGWG = Variation between genotypes within groups; VWG = Variation within genotypes; TV = Total variation.

### Representative subsets of the collection

Out of the total of 105 Napier grass accessions, 84 (60 from the ILRI collection and 24 from EMBRAPA) are currently being evaluated in the field for nutritional value, agronomic performance and water use efficiency under optimal-water and water-deficit conditions. Accession ILRI_16621, which had very high missing values out of the 60 accessions from ILRI, as well as 15 elite lines from the EMBRAPA collection were excluded from further analysis. The remaining 68 Napier grass accessions, comprising 59 from ILRI and nine from EMBRAPA that have both phenotype and genotype data, were selected and used to construct a core subset. The initial phenotypic trait data (Supplementary Table S6) were used to complement the selected 980 genome-wide SNP marker data in the analysis. UPGMA analysis clustered the 68 accessions into seven sub groups (Supplementary Fig. S5), and each subgroup was well represented in the subsets. Forage biomass traits, total fresh weight per plant (TFWPP) and total dry weight per plant (TDWPP), were highly variable among accessions in the subgroups. Groups II and IV had higher mean values while groups I and VII had lower mean values for both traits under optimal-water conditions. A similar trend was observed under water-deficit conditions, except that group IV had an average mean value in this case (Supplementary Fig. S5).

A subset of 14 (20%) accessions representing the range of phenotypic and genetic diversity in the 68 Napier grass accessions was identified for both optimal-water and water-deficit conditions and seven accessions are common between the two subsets (Supplementary Fig. 5; Table 4).

**Table 4:** Napier grass subsets representing the diversity in the collection from the ILRI genebank.

Optimal_water				Water-deficit			
NAME	Species	Origin	Collection	NAME	Species	Origin	Collection
ILRI_1026*	*purpureum*	Burundi	ILRI	ILRI_1026*	*purpureum*	Burundi	ILRI
ILRI_16840*	*purpureum* × *glaucum*	Zimbabwe	ILRI	ILRI_14389	*purpureum*	Nigeria	ILRI
ILRI_14982	*purpureum* × *glaucum*	USA	ILRI	ILRI_14983	*purpureum*	USA	ILRI
ILRI_14984	*purpureum*	USA	ILRI	ILRI_16811	*purpureum*	USA	ILRI
ILRI_16793*	*purpureum*	Cuba	ILRI	ILRI_16791	*purpureum*	Swaziland	ILRI
ILRI_16794	*purpureum*	Mozambique	ILRI	ILRI_16793*	*purpureum*	Cuba	ILRI
ILRI_16814*	*purpureum*	USA	ILRI	ILRI_16816	*purpureum*	USA	ILRI
ILRI_16839	*purpureum*	Zimbabwe	ILRI	ILRI_16796	*purpureum*	Zimbabwe	ILRI
ILRI_16819	*purpureum*	USA	ILRI	ILRI_16806*	*purpureum*	USA	ILRI
ILRI_16797	*purpureum*	Zimbabwe	ILRI	ILRI_16782	*purpureum*	Tanzania	ILRI
ILRI_16806*	*purpureum*	USA	ILRI	ILRI_16814*	*purpureum*	USA	ILRI
ILRI_16822	*purpureum*	Malawi	ILRI	ILRI_16840*	*purpureum* × *glaucum*	Zimbabwe	ILRI
BAGCE_30*	*purpureum*	Brazil	EMBRAPA	BAGCE_30*	*purpureum*	Brazil	EMBRAPA
BAGCE_97*	*purpureum*	Brazil	EMBRAPA	BAGCE_97*	*purpureum*	Brazil	EMBRAPA

*Accession selected in both subsets.

For a subset representing the overall diversity under optimal-water conditions, initial phenotype data of total fresh weight per plant (TFWPP) and total dry weight per plant (TDWPP) were used to complement the genome-wide SNP marker data (Table 5). Both the genetic and phenotypic diversity in the subset was similar to, or comparable with, the whole collection (Table 5). The average genetic distance using the modified Rogers (MR) distance of the subset was 0.46, which is higher than the value of 0.20 calculated for the whole collection. Similarly, the average phenotypic distance according to Gower distance (GD) was higher than the one in the whole collection. The remaining diversity indices, such as Shannon’s allelic diversity index (SH), expected heterozygosity (He) and polymorphic information content (PIC) were comparable between the subset and the whole collection.

**Table 5:** Comparisons between Napier grass accessions in the whole collection and the subsets for genetic and phenotypic diversity.

Trait	Whole collection			Subset (OW)			Subset (WD)		
	Min	Max	Average	Min	Max	Average	Min	Max	Average
EN-MR	0.20	0.21	0.20	0.32	0.48	0.46	0.35	0.48	0.46
EN-GD	0.08	0.09	0.08	0.12	0.22	0.19	0.11	0.22	0.19
Se	7.51	7.52	7.52	7.49	7.52	7.51	7.50	7.52	7.51
He	0.44	0.44	0.44	0.41	0.44	0.44	0.42	0.44	0.44
PIC	0.23	0.38	0.36	0.17	0.38	0.35	0.17	0.38	0.35
TFWPP	4.55 (37.01)	434.76 (313.16)	239.40 (139.71)	13.78	416.31	275.38	47.71	266.45	147.67
TDWPP	1.70 (7.92)	127.17 (87.85)	65.01 (39.86)	3.29	117.29	73.27	12.06	73.15	42.35
Fv/Fm	0.56	0.77	0.73	—	—	—	0.61	0.75	0.70
PI	1.09	5.37	2.86	—	—	—	1.11	4.82	2.62

EN-MR = Average entry-to-nearest-entry distance according to the Modified Rogers (MR) distance using the genetic data; EN-GD = Average entry-to-nearest-entry distance according to Gower distance (GD) using the phenotype data; SH = Shannon’s allelic diversity index; He = expected heterozygosity; PIC = polymorphic information content; TFWPP = total fresh-weight per plant; TDWPP = total dry-weight per plant; FvFM = the ratio of variable fluorescence to maximum fluorescence; PI = performance index. The phenotype of the whole collection under water-deficit conditions is in parentheses. OW = optimal-water condition; WD = water-deficit condition.

**Figure 5:**
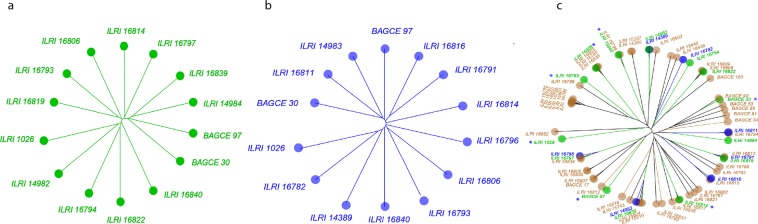
UPGMA tree for the subsets under optimal-water (**a**) and water-deficit (**b**) conditions. In (**c)**, the positions of the subsets in the whole collection (68 accessions) is shown by different colours, accessions not selected for the subsets are shaded a tan-color. Accessions common to the two subsets are indicated with asterisks.

The subset representing the genetic diversity under water-deficit conditions has a similar genetic and phenotypic diversity to the subset representing the optimal-water conditions (Fig. 5). However, additional phenotypes such as the ratio of variable fluorescence to maximum fluorescence (Fv/Fm) and performance index (PI) are included in this analysis (Supplementary Table S6). The Fv/Fm represents the plants maximum quantum efficiency of photosystem II (PSII) while PI represents the overall performance of photosynthesis. Both measure the level of plant stress and photosynthesis efficiency^43^.

## Discussion

Napier grass genetic characterization to date has relied mainly on assessing phenotypic traits^38^ and using low density molecular markers^22,24,36,44,45^ that are a poor proxy for the whole-genome information. In additions, most of the markers were selected based on cross-species transferability from closely related species^22,36^. Recent advances in genotyping by sequencing (GBS) approaches provide a cost-effective method for the identification of genome-wide molecular markers^31,33,46^ in species with non-existent or limited genomic information, such as Napier grass. Recently genome-wide SSR and SNP markers, based on transcriptome sequencing of Napier grass, have been reported^25,31^. In this study, we assessed the genetic diversity, population structure, and genome-wide patterns of linkage disequilibrium (LD) in the Napier grass collection maintained in the ILRI forage genebank and a collection acquired from EMBRAPA using genome-wide high-density SNP and SilicoDArT markers derived from the GBS method of the DArTseq platform, which combines genome complexity reduction using restriction enzymes and next generation sequencing^32,33^.

As a reference genome sequence remains to be generated for Napier grass, we used the pearl millet (*Pennisetum glaucum*) genome sequence to identify the genomic position and genome-wide distribution of the SilicoDArT and SNP markers. Pearl millet (2n = 2x = 14 chromosomes and with AA genomes) is closely related to Napier grass (2n = 4x = 28 chromosomes and genomes A′A′BB) and their genetic proximity allows the production of hybrids (2n = 3x = 21)^47^. In addition, the genome A of pearl millet and A′ of Napier grass are homeologs forming a pair in the hybrids during meiosis^47,48^. Furthermore, Napier grass sequences have been shown to display a high degree of synteny and considerable collinearity with the pearl millet genome^31^. The mapping showed the density and distribution of the markers across the genome. In addition, the mapping revealed that most of the markers are located at the peripheral ends of the chromosome arms, which is in line with many previous reports on other species including pearl millet^34,35,49^. However, we were only able to map about 17% of the SilicoDArT markers and 33% of the SNP markers to the seven chromosomes of the pearl millet genome, and very few of both marker types were mapped to different scaffolds, leaving more than 70% of the markers unmapped. This result is a little lower in comparison to the findings by Paudel *et al*.^31^, in which 38.8% of the Napier grass Illumina reads mapped onto the pearl millet genome. This is possibly due to differences in sequence read lengths produced by the Illumina and DArTseq platforms, as the former produces longer sequence reads. In addition, most of the unmapped markers could be from the Napier grass B genome which is not present in pearl millet^47^. The map position information was required to select a few representative, highly polymorphic and independent markers from across the genome for the genetic diversity analysis. The map position information was also required for a pair-wise LD calculation and for the genome-wide LD and LD-decay estimation. Furthermore, the map position information will be important for marker-trait association analysis and would aid in the identification of genomic regions controlling economically important traits in Napier grass.

### Genome-wide LD pattern in Napier grass

Information regarding the extent of genome-wide LD and LD-decay is important for genome-wide association studies (GWAS) that aim to detect linked markers and QTL affecting important traits, for the implementation of genomic selection and to support the design of genetic and plant breeding research strategies^50,51^. The distance over which LD persists determines the density of markers required for a GWAS analysis^52^. In this study, LD was analyzed between SilicoDArT markers with genomic position information based on the physical distance of the pearl millet reference genome.

LD was analyzed between pairs of markers on each chromosome and then combined to estimate the average LD-decay across the A′ genome in three Napier grass populations: the collection maintained at ILRI; the EMBRAPA collection, and; the combined population. We found that in the combined population LD decayed very rapidly and the value of *r*^2^ decreased to 0.2 at about 2.54 kbp. This fast rate of LD-decay could be due to the nature of Napier grass, which is a highly variable, heterozygous and cross-pollinating species. However, the variability and heterozygosity are fixed and maintained by the common practice of vegetative propagation through stem cuttings, in which meiosis and crossing over do not occur. The LD-decay estimated in this study was higher than that of pearl millet^34^ and foxtail millet^50^ but lower than that of sorghum^51,53^ and rice^54^, which are self-pollinating species. LD decays more rapidly in cross-pollinating compared to self-pollinating species, in which recombination is less effective^52^. The size of the Napier grass A′ genome has been estimated to be about 1.3 Gbp^31,47^, indicating that this about 24% smaller than the 1.7 Gbp pearl millet genome^34^. Taking the estimated genome size and the 2.54 kbp LD-decay into account, approximately 500,000 markers distributed across the genome would be required to detect QTL in the combined Napier grass population. This means that the current 116,190 silicoDArT markers roughly represent about 23% of the required markers. However, this percentage is likely to be an overestimate, if one considers the uneven distribution and the distance between markers.

LD decayed more slowly in the ILRI collection than the EMBRAPA material (Fig. 3), possibly reflecting the difference in breeding history of the two collections. Most of the Napier grass accessions in the ILRI genebank come from histroical collections or are older breeding lines from the USA which have been vegetatively propagated while maintained in the forage genebank. Conversely, most of the EMBRAPA accessions have passed through the Napier grass active breeding program and include elite lines developed during the breeding process. The very rapid rate of LD-decay observed in the EMBRAPA elite lines compared to the EMBRAPA collection further supports the effect of the breeding process on LD and suggests that a substantial reduction of LD has been achieved by the Napier grass active breeding program at EMBRAPA. The slower rate of LD-decay observed in the ILRI collection may indicate the presence of long haplotype blocks and suggest the presence of considerable variation in the ILRI material, the release of which will be important to capture more of the Napier grass diversity through breaking down the associations by crossing.

The rate of LD-decay varied across chromosomes in the ILRI and EMBRAPA populations as well as in the combined population. In most cases, the LD decayed fastest in chromosomes 6 and 3 while it was slowest in chromosome 1, implying that different marker densities will be required across the chromosomes for the application of GWAS in Napier grass. It also implies that a higher mapping resolution is expected for GWAS in the genomic regions with a fast rate of LD decay. It is quite common to see variation in the extent of LD across chromosomes^54^, mainly due to variation in recombination rate across the genome^55^. The information on the extent of genome-wide LD may serve as an important foundation for future applications of GWAS and marker-assisted selection in Napier grass.

### Genetic diversity and population structure

Genetic diversity and population structure analyses revealed the existence of a substantial amount of variation in the collections. In the analysis, we used selected SNP and SilicoDArT markers that were highly polymorphic, independent and distributed across the genome, which makes this report the first of its type and more robust than those reported previously using lower density marker sets. The presence of two to seven groups was observed by STRUCTURE, PCA and phylogenetic analyses and most of the materials from ILRI and EMBRAPA grouped separately. Analysis of molecular variance (AMOVA) indicated that the seven groups detected are significantly different from each other, with up to 14% variation among the groups. The high level of diversity and population stratification observed could be attributed to the outcrossing, self-incompatibility^20^ and polyploid nature of Napier grass. Furthermore, selection, breeding systems, and variation in geographical origin may also be contributing to the variation seen between the materials from ILRI and EMBRAPA. The impact of genetic drift and gene flow on the genetic variation is expected to be low as Napier grass is predominantly propagated clonally through stem cuttings and because of its reported low seed set and germination rate^45,56^. Another possible reason for the high diversity observed in the present study may be associated with the rich gene pool of the genus *Pennisetum* in general and the wide parental diversity of Napier grass in particular^36^. According to Robert *et al*.^57^, the genus has three gene pools: the primary gene pool consists of domesticated and wild weedy forms of *P. glaucum*; the secondary gene pool includes perennial and wild relatives of *P. purpureum* and *P. squamulatum*, which easily cross with *P. glaucum* but produce sterile hybrids; and the tertiary gene pool is comprised of true biological species which includes more than fifty species^57^.

The clustering within the materials from ILRI and EMBRAPA did not appear to be based on geographical origin, which is in line with the findings by Negawo *et al*.^24^ and Kandel *et al*.^22^ but contradicts the report by Harris *et al*.^40^ and Lowe *et al*.^12^. The EMBRAPA genotypes are mainly found in Groups I, II, and III; Group III being purely elite lines while Groups I and II contain mainly elite lines and genebank materials, which might reflect the breeding history of the elite lines. There is not enough information available to correlate the grouping with the pedigree of the accessions in a robust manner. However, based on the pedigree information provided by Harris *et al*.^40^, some of the accessions in Groups IV and VII (Fig. 4a) are derived from Merkeron, which is a Napier grass cultivar derived from an intraspecific cross between a high yielding clone and a dwarf leafy clone, with improved yield and disease resistance^41^ and has been used as a parental line at the breeding program in Tifton, Georgia^40^.

Clustering of the EMBRAPA elite lines could also be attributed to their pedigree history, as most genotypes within a cluster share a common parent (Supplementary Table S5). The eight *P. purpureum* × *P. glaucum* hybrids did not cluster together, but were distributed across Groups IV, V, and VI, which is a finding that is consistent with the previous report using SSR markers^29^. The diversity and population stratification of the Napier grass collections identified in this study are key findings which can be used as a guide for the effective management, utilization and improvement of the accessions, as well as in designing QTL mapping experiments.

### Sub-setting Napier grass accessions

The accessions selected as subsets in this study are few, but they well represent the overall genetic and phenotypic diversity of the collections held in the ILRI genebank. In addition, they are of a manageable size for distribution by the genebank and evaluation by agronomists, extension agents, non-governmental organizations (NGOs) or researchers in national research institutes, in different production systems and agro-ecological conditions. Screening and evaluation of the whole germplasm collection for target traits would be time-consuming, laborious, and costly. Hence the subsets serve as reference sets, representing the genetic diversity of the whole collection, they provide an entry point to the whole collection and improve access to the germplasm collection for plant breeders, researchers and other users^58^. Further to this, some of the accessions in the subsets are diverse at both the genotypic and phenotypic level and could offer the opportunity to identify heterotic groups for Napier grass improvement. The greater the genetic difference between the parents, the higher the level of heterosis, which is the phenomenon in which the progeny from hybridization display enhanced production traits when compared to the parents^59^.

Currently, more than 105 Napier grass accessions collected from across a range of environments and origins are maintained at the ILRI field sites at Bishoftu and Ziway (Batu) in Ethiopia. This conservation method could be prone to threats from natural disasters, such as pests, diseases and earthquakes, and/or anthropogenic disasters such as political unrest which is a common occurrence in third world countries. Therefore, establishing a representative subset of the whole collection also offers an additional opportunity for the conservation, management and use of the diversity held in the collection as the subsets provide a backup for conservation at different sites and in different countries.

### Candidate genes

Comparative DNA sequence analysis with closely related species, based on sequence similarity and genomic position of markers or short sequences, is a powerful approach to identifying candidate genes. The identification of candidate genes help us gain a better understanding of the evolution of species and determine the function of genes and non-coding regions in the genome^60,61^. In this regard, the availability of reference sequences and genomic information from the closely related species of pearl millet and foxtail millet^34,35^ have provided an important resource for genetic and genomic studies in Napier grass, a species with very little sequence information available. Comparative genomics can also be used to locate desirable alleles known in pearl millet or in Napier grass so that transfer can be achieved by conventional breeding as the two species can interbreed^24,62^. The recently reported genetic linkage map of Napier grass^31^ offers an additional resource to move forward our Napier grass genetic studies and for the identification of candidate genes and DNA markers to be used in marker assisted breeding.

We mapped 28,610 SNP and 20,144 SilicoDArT markers produced by the DArTseq platform on to the reference genome of pearl millet (*P. glaucum*)^34^ and generated chromosomal locations for these markers. The location was used to identify the closest gene aligned with the markers and the corresponding annotation information used to label 2,256 of the SNP markers. Although the majority of the annotation queries were uncharacterized proteins, there were some markers that aligned with known genes involved in economically important traits such as lipoxygenase^63^ and mitogen-activated protein kinase^64^ which are associated with disease resistance, and, trehalose 6-phosphate^65^, transcription elongation factor^66^ and auxin efflux carrier protein^67^ which are involved in drought tolerance.

In addition, the 2,256 SNP markers with map-positions and annotation information were compared with a list of pearl millet genes which have been associated with important traits by GWAS during a previous study^34^. Twenty-two of the markers shared sequence identity with genes associated with important traits, including fresh stover yield, plant height and plant population density in pearl millet (Table 2). These genes offer interesting candidates to be tested for association with these traits in Napier grass. For example, raffinose synthase, ubiquitin-associated/translation elongation factor and heat shock proteins have been associated with plant height under optimal-water conditions. The transcription factor-SBP-box has been associated with plant population density under late stress conditions while the ionotropic glutamate receptor is associated with fresh stover yield in early stress conditions^34^.

Although these findings are preliminary, decades of breeding work and molecular analysis of pearl millet can be exploited and future in-depth comparative genomic analyses between the two species offers and opportunity to leverage the resources available in pearl millet to support the improvement of Napier grass in the future.

## Conclusions

1. Genotyping by sequencing (GBS) using the DArTseq platform generated high-density and genome-wide distributed SilicoDArT (dominant) and SNP (co-dominant) markers, which are heterozygous, polymorphic and suitable for genetic and molecular diversity studies as well as for marker-trait association analysis. The SilicoDArT and SNP markers identified in our study, in conjunction with SSR and SNP markers developed by Paudel *et al*.^31^ and Wang *et al*.^25^, serve to enhance the data resources available for Napier grass improvement using marker assisted breeding.
2. The genetic diversity analysis revealed the presence of considerable variation in the Napier grass collection maintained in the ILRI genebank and identified some unique materials from the EMBRAPA collection, showing the suitability of the population for further genetic and marker-trait association studies.
3. A fast rate of LD-decay was observed across the Napier grass A′ genome and the LD decayed more slowly in the ILRI collection when compared to the EMBRAPA collection, suggesting that the materials from ILRI contain long haplotype blocks, in which a large amount of variation may be stacked which could potentially be released by crossing.
4. The availability of the pearl millet reference genome is an important asset for comparative DNA sequence analysis between pearl millet and Napier grass and is a resource for the identification of candidate genes associated with important forage traits. The candidate genes which have been shown to be associated with important forage traits in pearl millet need to be assessed and validated in Napier grass.
5. Subsets of Napier grass accessions that represent the genetic and phenotypic diversity held in the collections maintained in the ILRI genebank have been identified. These subsets are of a manageable size and act as a reference set for distribution and evaluation in different agro-ecologies and production systems.

## Methods

### Plant materials and DNA extraction

A Napier grass population comprising of 105 accessions assembled from the ILRI genebank and EMBRAPA collections and maintained at the Bishoftu and Ziway (Batu) sites in Ethiopia was used in the study. Leaf tissues were collected, freeze-dried and total DNA was extracted using a DNeasy plant mini kit (250) (Qiagen Inc., Valencia, CA) according to the manufacturer’s instructions.

DNA quality was measured on a Nanodrop (DeNovix DS-11 FX spectrophotometer) and a further quality check was carried out by agarose (0.8%) gel electrophoresis with 50 ng of lambda DNA as a marker. Samples were diluted to obtain the required concentration range (50–100 ng/µl) for the DArT genotyping platform. Once standardized, 25 µl of each sample was aliquoted into 96 well semi-skirted plates, packaged and shipped for genotyping.

### Genotyping by the DArTseq platform

Genotyping was carried out by Diversity Array Technology (http://www.diversityarrays.com/) using the DArTseq platform^33^ that combines genome complexity reduction using a combination of restriction enzymes and next-generation sequencing. Approximately 50 ng of genomic DNA was digested with a combination of *PstI/HpaII* restriction endonucleases and the resulting fragments were ligated to a *PstI* overhang compatible oligonucleotide adapter and sequenced on an Illumina HiSeq 2500 (Illumina) using *PstI* site-specific primers. Short sequence fragments, SilicoDArT (presence/absence), and SNP markers were generated following the DArTseq protocol. The short sequence fragments were aligned with the pearl millet (*Pennisetum glaucum*) reference sequence (http://cegsb.icrisat.org/ipmgsc/genome.html) to generate information on map position of the sequences and markers across the genome. The synbreed R-package^68^ was used for graphical representation and to visualize the density and genome-wide distribution of the markers across the genome.

### SNP annotation

For annotation, the genomic information resources of *P. glaucum* and *Setaria italica* were used. The transcribed genome of *P. glaucum* was blasted against the *S. italica* transcribed genome database and vice versa by the technique of reciprocal blastx, using the NCBI’s BLAST tool (https://blast.ncbi.nlm.nih.gov/Blast.cgi?PROGRAM=blastx&PAGE_TYPE=BlastSearch&BLAST_SPEC=&LINK_LOC=blasttab&LAST_PAGE=blastp). The two BLAST results (*P. glaucum* to *S. italica* and *S. italica* to *P. glaucum*) with best scores (a BLASTP Expect value of less than 10) were joined using the ‘subject’ and ‘query’ fields (being gene identifiers) and a set of 18,996 reciprocal blast best hits was produced. The annotation information for *S. italica* was extracted using UniProt (free-text gene function and Gene Ontology annotations) and merged with the association list, which was in turn matched with the SNPs based on their genomic position. SNPs with map-positions and annotation information were compared with pearl millet genes detected in a previous GWAS study^34^ and used for candidate gene selection by a reciprocal best hit BLAST analysis (i.e. when the best hit for protein A in pearl millet is protein B in foxtail millet, and the best hit for B in foxtail millet is the original protein A in pearl millet), which resulted in the generation of a total of 18,996 best hits.

### Marker data analysis

The percentage of missing data and minor allele frequency (MAF) per marker and per genotype were calculated in Microsoft Excel. Expected heterozygosity (He) and polymorphic information content (PIC) were calculated using locally written scripts in R statistical software (https://www.r-project.org/). The PIC value for each marker was calculated using the following formula^69^:

$${\rm{PIC}}=1-\sum _{i=1}^{l}{P}_{i}^{2}-\sum _{i=1}^{l-1}\sum _{j=i+1}^{l}2{P}_{i}^{2}{P}_{j}^{2}\,$$

### Linkage disequilibrium (LD) analysis and construction of LD-decay plots

Pair-wise LD using the correlation coefficient (*r*^2^) was estimated for pairs of SilicoDArT markers with known genomic locations based on the alignment with the pearl millet reference genome. The LD was estimated only for pairs of SilicoDArT markers located on the same chromosome. The markers with less than 10% missing values and a minor allele frequency higher than 5% were used in the LD analysis. For all pairs of SilicoDArT markers per chromosome, *r*^2^ values were calculated using PLINK v1.09^70^ (https://www.cog-genomics.org/plink2).

The pattern and distribution of intra-chromosomal LD were visualized and studied from LD plots generated for each chromosome by locally written R-scripts. To investigate the average rate of LD decay across the whole genome, the *r*^2^ values from all chromosomes were pooled and plotted against the physical distance between markers. Curves of rate of LD decay plotted against physical map distances were fitted by nonlinear regression, where the expectation of *r*^2^ between adjacent sites was determined.

### Diversity analysis

Population structure in the Napier grass population was estimated using selected independent SNP markers distributed across the genome, based on the pearl millet reference genome. The selected SNP markers had a MAF ≥ 10%, missing values ≤ 10% and a proximal marker-to-marker *r*^2^ value ≤ 0.5. A Bayesian clustering approach implemented in the STRUCTURE software^71^ was used to assess population structure. The burn-in time and number of iterations were both set to 100,000 with 10 repetitions, testing the probability of 20 subpopulations in an admixture model with correlated allele frequencies. The results of the run were uploaded to the software “Structure Harvester”^72^ (http://taylor0.biology.ucla.edu/structureHarvester/) and the most likely number of subpopulations was determined by the Evanno method^73^.

In addition, population structure was estimated by a principal component analysis (PCA) with the selected independent SNP markers distributed across the genome using the R-package adegenet^74^. An unweighted pair-group mean arithmetic (UPGMA) tree was created to visualize relationships between genotypes using the R package Analyses of Phylogenetics and Evolution (ape)^75^. Cophenetic correlation and correlation analyses using the R package dendextend^42^ were used to determine how well the phylogenetic tree represented the original distance matrix. Furthermore, an analysis of molecular variance (AMOVA) was performed to estimate the variance among populations detected by the above described methods and among genotypes within populations using the R package poppr^76^.

### Field phenotyping of Napier grass accessions

A collection of 84 (60 ILRI collection and 24 EMBRAPA) Napier grass genotypes were planted in an augmented p-rep design with four replications. Six stem cuttings per accession were planted in a single row with a distance of 750 mm between plants. Approximately three months after establishment in the main rainy season (mid-June to mid-September, 2017), the plants were clean cut to a standard height of 50 mm above ground. A drought stress experiment was initiated in the dry season at the beginning of 2018 where two blocks of Napier grass plants were irrigated to a volumetric soil water content (VWC) of 20% i.e. optimal water (OW) and the other two blocks were irrigated with a reduced amount of water which corresponds to a VWC of 10% i.e. water stress (WS). The soil water content of both watering regimes was monitored using a Delta soil moisture probe (HD, England). Following every 8 weeks of regrowth, plants were cut to a height of 50 mm and total fresh weight per plant (TFWPP) was taken by weighing and calculating the average from three randomly selected plants per row. Total dry weight per plant (TDWPP) was estimated from oven-dried samples (65 °C for 72 h) by taking 600 g from each fresh weight sample. Chlorophyll fluorescence was measured at the middle part of the abaxial side of the third leaf from the top after dark-adaptation for 20 min with an *in situ* portable fluorometer, Pocket Plant Efficiency Analyzer (PEA) (Hansatech, King’s Lynn, Norfolk, UK). The chlorophyll fluorescence parameters measured were the efficiency of excitation energy captured by open PSII reaction (Fv/Fm) and the performance index (PI) which measures the overall force of the light and dark reactions. The average values from three harvests were used in the diversity analysis to support the selection of Napier grass subsets. An averaged data value per trait per accession was generated for each of the conditions. The averaged data value was calculated based on 18 plants per accession recorded from 3 plants per row, in two replications, across three harvests collected in 2018, for each of the OW and WD conditions. The averaged values were used in further diversity analysis for the selection of Napier grass subsets.

### Sub-setting Napier grass genotypes representative of the population

To select a subset of representative accessions, the R package Core Hunter v. 3.2.1^15^ was used. This program is able to identify core subsets using diverse allocation strategies by optimizing many genetic parameters simultaneously. The modified Roger’s distance (RD), Shannon’s information index (SH), average entry-to-nearest-entry distance (EN), expected proportion of heterozygous loci (He) and allele coverage (CV), each with an equal weight, were used to define a core subset representing the entire collection. In addition, for the targeted subsets an initial analysis of TFWPP and TDWPP were considered together with the genetic information. Additional phenotypes, such as the ratio of variable fluorescence to maximum fluorescence (Fv/Fm) and performance index (PI) were used under water deficit conditions. The ILRI genebank accessions are freely available to researchers who accept the terms and conditions of the Standard Material Transfer Agreement (SMTA) of the International Treaty on Plant Genetic Resources for Food and Agriculture (http://www.fao.org/planttreaty/areas-of-work/the-multilateral-system/the-smta/en/)^77–79^.

## Supplementary information

Supplementary Tables and Figures

Supplementary Tables S2, S3 and S4

## Data Availability

Most of the datasets generated in the current study are found in supplementary information and additional data are available from the corresponding author on reasonable request.
