# Author Correction: Genotyping by sequencing provides new insights into the diversity of Napier grass (*Cenchrus purpureus*) and reveals variation in genome-wide LD patterns between collections

**DOI:** 10.1038/s41598-020-62001-2

**Published:** 2020-03-13

**Authors:** Meki S. Muktar, Abel Teshome, Jean Hanson, Alemayehu T. Negawo, Ermias Habte, Jean-Baka Domelevo Entfellner, Ki-Won Lee, Chris S. Jones

**Affiliations:** 1 Feed and Forage Development, International Livestock Research Institute, Addis Ababa, Ethiopia; 2 Teagasc|CELUP Crop Research, Oak Park, Carlow, R93 XE12 Ireland; 3 Grassland and Forages Division, National Institute of Animal Science, Rural Development Administration, Cheonan, 31000 Republic of Korea; 4 Biosciences Eastern and Central Africa, International Livestock Research Institute, Nairobi, Kenya; 5 Feed and Forage Development, International Livestock Research Institute, Nairobi, Kenya

Correction to: *Scientific Reports* 10.1038/s41598-019-43406-0, published online 06 May 2019

This Article contains errors.

In the Results section, under the subheading ‘Representative subsets of the collection’,

“A subset of 14 (20%) accessions representing the range of phenotypic and genetic diversity in the 68 Napier grass accessions was identified for both optimal-water and water-deficit conditions and seven accessions are common between the two subsets (Supplementary Fig. 5; Table 4).”

should read:

“A subset of 14 (20%) accessions representing the range of phenotypic and genetic diversity in the 68 Napier grass accessions was identified for both optimal-water and water-deficit conditions and seven accessions are common between the two subsets (Fig. 5; Table 4).”

In the legend for Table 5,

“FvFM = the ratio of variable fluorescence to maximum fluorescence”

should read:

“Fv/Fm = the ratio of variable fluorescence to maximum fluorescence”

In the Methods section, under the subheading ‘Field phenotyping of Napier grass accessions’,

“A drought stress experiment was initiated in the dry season at the beginning of 2018 where two blocks of Napier grass plants were irrigated to a volumetric soil water content (VWC) of 20% i.e. optimal water (OW) and the other two blocks were irrigated with a reduced amount of water which corresponds to a VWC of 10% i.e. water stress (WS).”

should read:

“A drought stress experiment was initiated in the dry season at the beginning of 2018 where two blocks of Napier grass plants were irrigated to a volumetric soil water content (VWC) of 20% i.e. optimal water (OW) and the other two blocks were irrigated with a reduced amount of water which corresponds to a VWC of 10% i.e. water-deficit (WD).”

In the same section,

“Total dry weight per plant (TDWPP) was estimated from oven-dried samples (65 °C for 72 h) by taking 600 g from each fresh weight sample. Chlorophyll fluorescence was measured at the middle part of the abaxial side of the third leaf from the top after dark-adaptation for 20 min with an in situ portable fluorometer, Pocket Plant Efficiency Analyzer (PEA) (Hansatech, King’s Lynn, Norfolk, UK). The chlorophyll fluorescence parameters measured were the efficiency of excitation energy captured by open PSII reaction (Fv/Fm) and the performance index (PI) which measures the overall force of the light and dark reactions.”

should read:

“Total dry weight per plant (TDWPP) was estimated from oven-dried samples (65 °C for 72 h) by taking 300 g from each fresh weight sample. Chlorophyll fluorescence was measured at the middle part of the abaxial side of the third leaf from the top after dark-adaptation for 20 min with an in situ portable fluorometer, Pocket Plant Efficiency Analyzer (PEA) (Hansatech, King’s Lynn, Norfolk, UK). The chlorophyll fluorescence parameters measured were the efficiency of excitation energy captured by open photosystem II (PSII) reaction (Fv/Fm) and the performance index (PI) which measures the overall force of the light and dark reactions.”

Finally, this Article contains errors in the Reference list. References 77 and 79 were erroneously included, and Reference 78 is a duplicate of Reference 19. As a result, in the Methods section, under the subheading ‘Sub-setting Napier grass genotypes representative of the population’,

“Additional phenotypes, such as the ratio of variable fluorescence to maximum fluorescence (Fv/Fm) and performance index (PI) were used under water deficit conditions. The ILRI genebank accessions are freely available to researchers who accept the terms and conditions of the Standard Material Transfer Agreement (SMTA) of the International Treaty on Plant Genetic Resources for Food and Agriculture (http://www.fao.org/planttreaty/areas-of-work/the-multilateral-system/the-smta/en/)^77,78,79^.”

should read:

“Additional phenotypes, such as the ratio of variable fluorescence to maximum fluorescence (Fv/Fm) and performance index (PI) were used under water-deficit conditions. The ILRI genebank accessions are freely available to researchers who accept the terms and conditions of the Standard Material Transfer Agreement (SMTA) of the International Treaty on Plant Genetic Resources for Food and Agriculture (http://www.fao.org/planttreaty/areas-of-work/the-multilateral-system/the-smta/en/).”

