# Supplementary material for: Genotyping by sequencing provides new insights into the diversity of Napier grass (Cenchrus purpureus) and reveals variation in genome-wide LD patterns between collections
Source: Sci Rep. 2019 May 6;9:6936. doi: 10.1038/s41598-019-43406-0 (PMC6502793; doi:10.1038/s41598-019-43406-0)
Supplement: Supplementary file 1 — Supplementary Tables and Figures [file 41598_2019_43406_MOESM1_ESM.pdf]

Title: Genotyping by sequencing provides new insights into the diversity of Napier grass (*Cenchrus purpureus*) and reveals variation in genome-wide LD patterns between collections

Authors: Meki S. Muktar[1*], Abel Teshome[2*], Jean Hanson[1], Alemayehu T. Negawo[1], Ermias Habte[1], J.-B. Domelevo Entfellner[4], Ki-Won Lee[3], and Chris S. Jones[5]

SUPPLEMENTARY FILES

Legend:

Supplementary Tables

Supplementary Table S1. Napier grass population used in the study

Supplementary Table S2. DArT SNPs annotated based on *P. glaucum* and *S. italica* sequence annotation information (See separate supplementary data file)

Supplementary Table S3. Selected SNPs for diversity analysis (See separate supplementary data file)

Supplementary Table S4. Selected SilicoDArTs for diversity analysis (See separate supplementary data file)

Supplementary Table S5. Pedigree information for the EMBRAPA elite lines

Supplementary Table S6. Mean phenotypic data used in subsetting

Supplementary Figures

Supplementary Figure S1. LD-decay in EMBRAPA-collection versus EMBRAPA-elite lines

Supplementary Figure S2. Genome-wide distribution, PIC and He of SNPs and SilicoDArTs selected for diversity analysis

Supplementary Figure S3. Comparisons of plots of original distance matrix versus recalculated distance for NJ, UPGMA, and hclust (hierarchical clustering)

Supplementary Figure S4. Comparisons in clustering between the SNPs and SilicoDArTs selected for diversity analysis

Supplementary Figure S5. Comparison of mean phenotypic variability among subgroups

SUPPLEMENTARY TABLES

Supplementary Table S1. Napier grass accessions used in the study

No.	Acc. No.	Genus	Species	Origin	Collection	DOI	Year acquired
1	ILRI_1026	Pennisetum	purpureum	Burundi	ILRI	10.18730/FPB6*	1986
2	ILRI_14355	Pennisetum	purpureum	Ethiopia	ILRI	10.18730/FSSQH	1985
3	ILRI_14389	Pennisetum	purpureum	Nigeria	ILRI	10.18730/FSTNA	1985
4	ILRI_14982	Pennisetum	purpureum x glaucum	USA	ILRI	10.18730/FT9VF	1986
5	ILRI_14983	Pennisetum	purpureum	USA	ILRI	10.18730/FT9WG	1986
6	ILRI_14984	Pennisetum	purpureum	USA	ILRI	10.18730/FT9XH	1986
7	ILRI_15357	Pennisetum	purpureum x glaucum	NA	ILRI	10.18730/FTMBJ	1986
8	ILRI_15743	Pennisetum	purpureum	USA	ILRI	10.18730/FTZF4	1988
9	ILRI_16621	Pennisetum	purpureum	Namibia	ILRI	10.18730/FVP6W	1991
10	ILRI_16782	Pennisetum	purpureum	Tanzania	ILRI	10.18730/FVTY*	1992
11	ILRI_16783	Pennisetum	purpureum	Tanzania	ILRI	10.18730/FVTZ~	1992
12	ILRI_16784	Pennisetum	purpureum	Tanzania	ILRI	10.18730/FVV0$	1992
13	ILRI_16785	Pennisetum	purpureum	Tanzania	ILRI	10.18730/FVV1=	1992
14	ILRI_16786	Pennisetum	purpureum	Swaziland	ILRI	10.18730/FVV2U	1992
15	ILRI_16787	Pennisetum	purpureum	Swaziland	ILRI	10.18730/FVV30	1992
16	ILRI_16788	Pennisetum	purpureum	Swaziland	ILRI	10.18730/FVV41	1992
17	ILRI_16789	Pennisetum	purpureum	Swaziland	ILRI	10.18730/FVV52	1992
18	ILRI_16791	Pennisetum	purpureum	Swaziland	ILRI	10.18730/FVV74	1992
19	ILRI_16792	Pennisetum	purpureum	Mozambique	ILRI	10.18730/FVV85	1992
20	ILRI_16793	Pennisetum	purpureum	Cuba	ILRI	10.18730/FVV96	1992
21	ILRI_16794	Pennisetum	purpureum	Mozambique	ILRI	10.18730/FVVA7	1992
22	ILRI_16795	Pennisetum	purpureum	Zimbabwe	ILRI	10.18730/FVVB8	1992
23	ILRI_16796	Pennisetum	purpureum	Zimbabwe	ILRI	10.18730/FVVC9	1992
24	ILRI_16797	Pennisetum	purpureum	Zimbabwe	ILRI	10.18730/FVVDA	1992
25	ILRI_16798	Pennisetum	purpureum	Zimbabwe	ILRI	10.18730/FVVEB	1992
26	ILRI_16799	Pennisetum	purpureum	Zimbabwe	ILRI	10.18730/FVVFC	1992
27	ILRI_16800	Pennisetum	purpureum	Zimbabwe	ILRI	10.18730/FVVHE	1992
28	ILRI_16801	Pennisetum	purpureum	Zimbabwe	ILRI	10.18730/FVVJF	1992
29	ILRI_16802	Pennisetum	purpureum	Zimbabwe	ILRI	10.18730/FVVKG	1992
30	ILRI_16803	Pennisetum	purpureum	Zimbabwe	ILRI	10.18730/FVVMH	1992
31	ILRI_16804	Pennisetum	purpureum	USA	ILRI	10.18730/FVVNJ	1992
32	ILRI_16805	Pennisetum	purpureum	USA	ILRI	10.18730/FVVPK	1992
33	ILRI_16806	Pennisetum	purpureum	USA	ILRI	10.18730/FVVQM	1992

34	ILRI_16807	Pennisetum	purpureum	USA	ILRI	10.18730/FVVRN	1992
35	ILRI_16808	Pennisetum	purpureum	USA	ILRI	10.18730/FVVSP	1992
36	ILRI_16809	Pennisetum	purpureum	USA	ILRI	10.18730/FVVTQ	1992
37	ILRI_16810	Pennisetum	purpureum	USA	ILRI	10.18730/FVVVR	1992
38	ILRI_16811	Pennisetum	purpureum	USA	ILRI	10.18730/FVVWS	1992
39	ILRI_16812	Pennisetum	purpureum	USA	ILRI	10.18730/FVVXT	1992
40	ILRI_16813	Pennisetum	purpureum	USA	ILRI	10.18730/FVVYV	1992
41	ILRI_16814	Pennisetum	purpureum	USA	ILRI	10.18730/FVVZW	1992
42	ILRI_16815	Pennisetum	purpureum	USA	ILRI	10.18730/FVW0X	1992
43	ILRI_16816	Pennisetum	purpureum	USA	ILRI	10.18730/FVW1Y	1992
44	ILRI_16817	Pennisetum	purpureum	USA	ILRI	10.18730/FVW2Z	1992
45	ILRI_16818	Pennisetum	purpureum	USA	ILRI	10.18730/FVW3*	1992
46	ILRI_16819	Pennisetum	purpureum	USA	ILRI	10.18730/FVW4~	1992
47	ILRI_16821	Pennisetum	purpureum	Zimbabwe	ILRI	10.18730/FVW5$	1992
48	ILRI_16822	Pennisetum	purpureum	Malawi	ILRI	10.18730/FVW6=	1992
49	ILRI_16834	Pennisetum	purpureum x glaucum	Zimbabwe	ILRI	10.18730/FVWJA	1992
50	ILRI_16835	Pennisetum	purpureum x glaucum	Zimbabwe	ILRI	10.18730/FVWKB	1992
51	ILRI_16836	Pennisetum	purpureum	Zimbabwe	ILRI	10.18730/FVWMC	1992
52	ILRI_16837	Pennisetum	purpureum x glaucum	Zimbabwe	ILRI	10.18730/FVWND	1992
53	ILRI_16838	Pennisetum	purpureum x glaucum	Zimbabwe	ILRI	10.18730/FVWPE	1992
54	ILRI_16839	Pennisetum	purpureum	Zimbabwe	ILRI	10.18730/FVWQF	1992
55	ILRI_16840	Pennisetum	purpureum x glaucum	Zimbabwe	ILRI	10.18730/FVWRG	1992
56	ILRI_16902	Pennisetum	purpureum	Zimbabwe	ILRI	10.18730/FVYN3	1992
57	ILRI_16790	Pennisetum	purpureum	Swaziland	ILRI	10.18730/FVV63	1992
58	ILRI_18438	Pennisetum	purpureum	Tanzania	ILRI	10.18730/FX7YX	1995
59	ILRI_18448	Pennisetum	purpureum	Tanzania	ILRI	10.18730/FX871	1995
60	ILRI_18662	Pennisetum	purpureum x glaucum	South_Africa	ILRI	10.18730/FXENP	2006
61	BABCE-1	Pennisetum	purpureum	Colombia	EMBRAPA_collection	NA	1976
62	BABCE-100	Pennisetum	purpureum	Brazil	EMBRAPA_collection	NA	1993
63	BABCE-16	Pennisetum	purpureum	Brazil	EMBRAPA_collection	NA	1976
64	BABCE-17	Pennisetum	purpureum	Costa Rica	EMBRAPA_collection	NA	1976
65	BABCE-22	Pennisetum	purpureum	NA	EMBRAPA_collection	NA	1976
66	BABCE-24	Pennisetum	purpureum	NA	EMBRAPA_collection	NA	1976
67	BABCE-25	Pennisetum	purpureum	India	EMBRAPA_collection	NA	1976
68	BABCE-30	Pennisetum	purpureum	Brazil	EMBRAPA_collection	NA	1976
69	BABCE-34	Pennisetum	purpureum	Brazil	EMBRAPA_collection	NA	1976
70	BABCE-53	Pennisetum	purpureum	Brazil	EMBRAPA_collection	NA	1976
71	BABCE-56	Pennisetum	purpureum	Brazil	EMBRAPA_collection	NA	1989
72	BABCE-63	Pennisetum	purpureum	Cuba	EMBRAPA_collection	NA	1991

73	BABCE-7	Pennisetum	purpureum	Brazil	EMBRAPA_collection	NA	1976
74	BABCE-75	Pennisetum	purpureum	Brazil	EMBRAPA_collection	NA	1992
75	BABCE-80	Pennisetum	purpureum	Brazil	EMBRAPA_collection	NA	1992
76	BABCE-81	Pennisetum	purpureum	Brazil	EMBRAPA_collection	NA	1992
77	BABCE-86	Pennisetum	purpureum	NA	EMBRAPA_collection	NA	1992
78	BABCE-90	Pennisetum	purpureum	NA	EMBRAPA_collection	NA	NA
79	BABCE-94	Pennisetum	purpureum	NA	EMBRAPA_collection	NA	1993
80	BABCE-97	Pennisetum	purpureum	NA	EMBRAPA_collection	NA	1993
81	CNPGL_00-1-1	Pennisetum	purpureum	NA	EMBRAPA_elite_lines	NA	NA
82	CNPGL_91-06-2	Pennisetum	purpureum	NA	EMBRAPA_elite_lines	NA	NA
83	CNPGL_91-11_-2	Pennisetum	purpureum	NA	EMBRAPA_elite_lines	NA	NA
84	CNPGL_91-25-1	Pennisetum	purpureum	NA	EMBRAPA_elite_lines	NA	NA
85	CNPGL_92-133-3	Pennisetum	purpureum	NA	EMBRAPA_elite_lines	NA	NA
86	CNPGL_92-198-7	Pennisetum	purpureum	NA	EMBRAPA_elite_lines	NA	NA
87	CNPGL_92-190-1	Pennisetum	purpureum	NA	EMBRAPA_elite_lines	NA	NA
88	CNPGL_92-38-2	Pennisetum	purpureum	NA	EMBRAPA_elite_lines	NA	NA
89	CNPGL_92-56-2	Pennisetum	purpureum	NA	EMBRAPA_elite_lines	NA	NA
90	CNPGL_92-66-3	Pennisetum	purpureum	NA	EMBRAPA_elite_lines	NA	NA
91	CNPGL_92-79-2	Pennisetum	purpureum	NA	EMBRAPA_elite_lines	NA	NA
92	CNPGL_93-01-1	Pennisetum	purpureum	NA	EMBRAPA_elite_lines	NA	NA
93	CNPGL_93-04-2	Pennisetum	purpureum	NA	EMBRAPA_elite_lines	NA	NA
94	CNPGL_93-06-1	Pennisetum	purpureum	NA	EMBRAPA_elite_lines	NA	NA
95	CNPGL_93-08-1	Pennisetum	purpureum	NA	EMBRAPA_elite_lines	NA	NA
96	CNPGL_93-18-2	Pennisetum	purpureum	NA	EMBRAPA_elite_lines	NA	NA
97	CNPGL_93-32-2	Pennisetum	purpureum	NA	EMBRAPA_elite_lines	NA	NA
98	CNPGL_92-37-5	Pennisetum	purpureum	NA	EMBRAPA_elite_lines	NA	NA
99	CNPGL_94-07-2	Pennisetum	purpureum	NA	EMBRAPA_elite_lines	NA	NA
100	CNPGL_94-13-1	Pennisetum	purpureum	NA	EMBRAPA_elite_lines	NA	NA
101	CNPGL_96-21-1	Pennisetum	purpureum	NA	EMBRAPA_elite_lines	NA	NA
102	CNPGL_96-23-1	Pennisetum	purpureum	NA	EMBRAPA_elite_lines	NA	NA
103	CNPGL_96-24-1	Pennisetum	purpureum	NA	EMBRAPA_elite_lines	NA	NA
104	CNPGL_96-27-3	Pennisetum	purpureum	NA	EMBRAPA_elite_lines	NA	NA
105	PIONEIRO	Pennisetum	purpureum	NA	EMBRAPA_elite_lines	NA	NA

NA = information not available

Supplementary Table S5. Pedigree information for the EMBRAPA elite lines

Group I

CNPGL_91-06-2	BAGCE 37 X BAGCE 49
CNPGL_92-190-1	BAGCE 49 x BAGCE 58 x (BAGCE 57 x BAGCE 58)
CNPGL_92-37-5	BAGCE 52 x BAGCE 58
CNPGL_93-04-2	(BAGCE 36 x BAGCE 37) x (BAGCE 49 x BAGCE 57)
CNPGL_93-18-2	(BAGCE 5 x BAGCE 38) x (BAGCE 57 x BAGCE 58)
CNPGL_93-08-1	(BAGCE 4 x BAGCE 11) x (BAGCE 57 x BAGCE 58)
CNPGL_92-56-2	BAGCE 11 x BAGCE 22

Group II

CNPGL_96-23-1	(BAGCE 3 x BAGCE 5) x BAGCE 75
CNPGL_96-27-3	(BAGCE 03 x BAGCE 5 -) = Pioneiro X BAGCE 25
CNPGL_96-24-1	BAGCE 37 x BAGCE 49
CNPGL_94-07-2	BAGCE 5 x BAGCE 38 x BAGCE 49 x BAGCE 57 x BAGCE 58
CNPGL_00-1-1	F1 of dwarf population
PIONIRO	BAGCE 3 x BAGCE 5

Group III

CNPGL_92-133-3	(BAGCE 9 x BAGCE **57**) x (BAGCE 58 x BAGCE 57)
CNPGL_91-25-1	BAGCE 57 x BAGCE 58
CNPGL_93-06-1	**BAGCE 57** x BAGCE 58
CNPGL_92-198-7	BAGCE 19 x **BAGCE 57**
CNPGL_94-13-1	BAGCE 38 x **BAGCE 57** x BAGCE 58 x and others
CNPGL_93-32-2	BAGCE 5 x BAGCE 38 x (**BAGCE 57** x BAGCE 58)

Supplementary Table S6. Mean phenotypic data used in subsetting

Genotype	Fv/Fm		PI		TFWPP(g/plant)		TDWPP(g/plant)	
	OW	WD	OW	WD	OW	WD	OW	WD
ILRI_1026	0.72	0.61	1.7	1.25	122.9	58.76	34.62	19.81
ILRI_14355	0.74	0.69	2.85	2.11	310.67	224.67	94.06	62.24
ILRI_14389	0.75	0.72	3.06	1.77	194.93	104.16	56.24	29.25
ILRI_14982	0.74	0.68	2.99	1.94	307.7	149.82	79.19	41.41
ILRI_14983	0.73	0.69	3.41	1.97	304.76	190.68	72.94	50.11
ILRI_14984	0.71	0.7	2.58	2.16	387.9	189.24	111.13	57.27
ILRI_15357	0.75	0.73	3.55	3.25	294.03	167.24	81.51	48.42
ILRI_15743	0.75	0.7	3.38	2.26	221.7	167.34	55.6	44.5
ILRI_16782	0.74	0.76	3.25	4.7	187.87	139.94	51.21	35.1
ILRI_16783	0.56	0.67	1.09	1.78	284.47	72.89	77.67	19.8
ILRI_16784	0.74	0.69	3.01	2.06	184.81	181.25	49.64	47.83
ILRI_16785	0.7	0.69	1.64	1.37	315.95	194.94	84.61	63.68
ILRI_16786	0.71	0.72	1.69	2.78	322.39	203.85	94.42	61.98
ILRI_16787	0.74	0.68	2.14	1.38	262.44	83.55	72.11	23.94
ILRI_16788	0.69	0.69	1.27	1.61	142.69	145.63	39.17	44.41
ILRI_16789	0.71	0.68	2.18	2.44	342.49	201.72	97.59	59.6
ILRI_16790	0.72	0.66	2.84	2.37	76.02	37.01	17.99	9.97
ILRI_16791	0.75	0.7	3.77	2.71	291.99	313.16	79.01	87.85
ILRI_16792	0.73	0.68	2.35	2.38	347.47	291.4	102.81	83.71
ILRI_16793	0.73	0.73	3.25	3.71	375.99	181.42	111.22	50.12
ILRI_16794	0.77	0.73	5.37	4.82	264.5	161.28	75.71	48.91
ILRI_16795	0.74	0.71	2.79	3.02	322.89	203.99	93.51	60.72
ILRI_16796	0.76	0.71	4.71	2.43	135.45	65.7	63.1	19.53
ILRI_16797	0.7	0.69	2.36	2.26	13.78	47.71	3.29	12.06
ILRI_16798	0.73	0.71	2.58	2.53	325.68	236.78	88.49	69.74
ILRI_16799	0.72	0.7	1.91	1.75	120.12	64.36	29.91	17.15
ILRI_16800	0.75	0.7	2.9	2.11	413.65	251.75	127.19	74.77
ILRI_16801	0.72	0.72	1.72	2.71	434.76	202.42	126.66	58.48
ILRI_16802	0.74	0.71	3.6	2.85	276.79	287.27	75.02	77.49
ILRI_16803	0.7	0.7	1.69	1.83	380.05	200.15	111.7	62.73
ILRI_16804	0.73	0.71	2.43	1.71	391.56	82.84	101.7	22.05
ILRI_16805	0.73	0.72	2.49	2.33	39.55	46.94	8.36	13.58
ILRI_16806	0.73	0.67	2.93	2.32	416.31	155.13	117.92	51.68
ILRI_16807	0.7	0.69	1.96	2.49	183.95	137.32	47.14	35.78
ILRI_16808	0.75	0.71	3.87	3.11	104.16	65.47	31.6	19.18
ILRI_16809	0.73	0.64	2.93	2.29	161	107.06	47.19	32.36
ILRI_16810	0.69	0.72	2.11	2.25	185	128.67	40.28	38.37

ILRI_16811	0.74	0.68	2.91	1.75	291.16	108.54	78.34	30.91
ILRI_16812	0.69	0.73	2.06	4.15	190.4	86	52.28	21.98
ILRI_16813	0.74	0.64	5.25	1.5	137.93	153.37	26.53	40.48
ILRI_16814	0.7	0.71	2.39	3.57	291.55	139.95	73.11	36.63
ILRI_16815	0.75	0.64	3.9	1.23	239.01	100.2	65.49	27.11
ILRI_16816	0.76	0.76	3.75	4.94	118.12	62.08	34.35	17.49
ILRI_16817	0.75	0.69	3.56	1.72	198.56	82.35	51.45	23.84
ILRI_16818	0.75	0.71	3.72	2.5	109.2	96.1	31.71	29.81
ILRI_16819	0.75	0.71	5.02	1.88	366.63	266.45	104.47	73.15
ILRI_16821	0.72	0.72	2.5	2.97	123.08	106.05	33.53	31.03
ILRI_16822	0.72	0.74	2.28	3.24	96.01	103.92	26.19	31.8
ILRI_16834	0.75	0.73	3.1	2.47	194.79	53.76	54.93	15.97
ILRI_16835	0.71	0.69	1.92	2.43	137.03	44.21	33.24	12.76
ILRI_16836	0.76	0.69	2.63	1.84	306.78	150.31	71.3	47.23
ILRI_16837	0.73	0.66	2.55	1.11	225.16	164.55	60.73	41.64
ILRI_16838	0.75	0.75	3.89	3.74	131.98	111.62	33.96	31.83
ILRI_16839	0.7	0.7	2.22	2.56	411.17	128.22	94.35	35.18
ILRI_16840	0.71	0.68	1.89	1.11	241.16	104.71	59.81	29.37
ILRI_16902	0.77	0.72	3.97	3.23	207.22	173.02	61.07	50.36
ILRI_18438	0.7	0.73	2.79	3.28	248.14	214.48	70.41	57.58
ILRI_18448	0.7	0.72	3.65	3.62	141.79	76.48	42.49	23.24
ILRI_18662	0.76	0.63	3.96	1.89	4.55	38.94	1.7	7.92
BAGCE_100	0.67	0.73	2.25	2.77	240.39	179.33	65.59	61.91
BAGCE_17	0.71	0.59	2.07	1.34	144.31	108.33	38.16	27.61
BAGCE_30	0.69	0.75	1.92	3.57	368.22	254.29	89.49	70.84
BAGCE_343	0.76	0.77	3.36	5.03	241.19	111.35	65.92	31.58
BAGCE_53	0.74	0.72	2.73	2.54	344.48	91.93	83.73	24.93
BAGCE_81	0.74	0.68	2.73	1.56	229.01	62.61	56.36	17.59
BAGCE_86	0.73	0.71	3.32	2.34	274.05	123.33	69.15	32.93
BAGCE_90	0.72	0.71	2.69	2.36	385.86	133.88	93.74	35.84
BAGCE_97	0.74	0.7	3.06	2.32	191.56	126.51	45.31	34.67

Fv/Fm =quantum efficiency of photosystem II, PI = performance index, TDWPP=total dry weight per plot, TFWPP= total fresh weight per plot.

SUPPLEMENTARY FIGURES

a. EMBRAPA_collection **b**. EMBRAPA_elite_lines

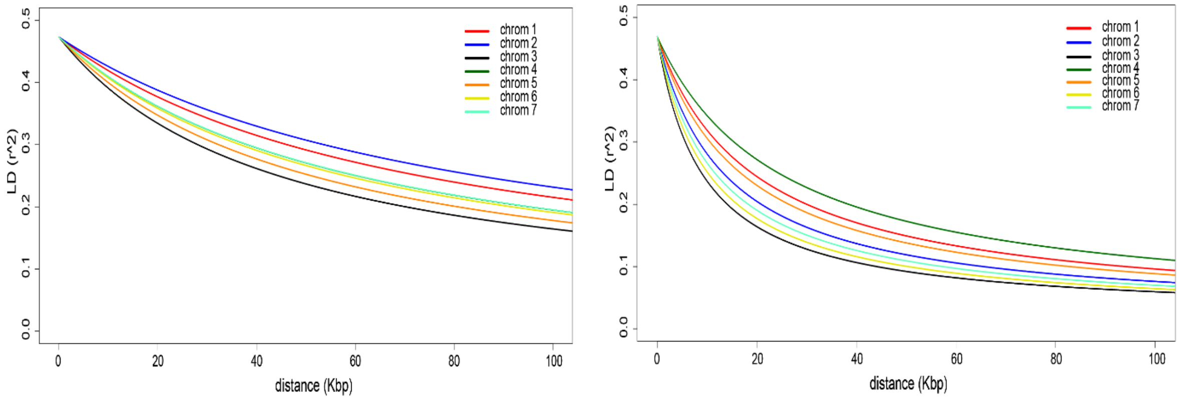

Supplementary Figure S1. The LD-decay in EMBRAPA collection (20 accessions) ranged from 50.13 kbp in chromosome 3 to 95.67 kbp in chromosome 2 (a), while in EMBRAPA elite lines (25 accessions) the LD-decay ranged from 10.35 kbp in chromosome 3 to 28.02 kbp in chromosome 4 (b). The average LD across the genome in the collection was 68.03 kbp, while it was 16.56 kbp in the elite lines.

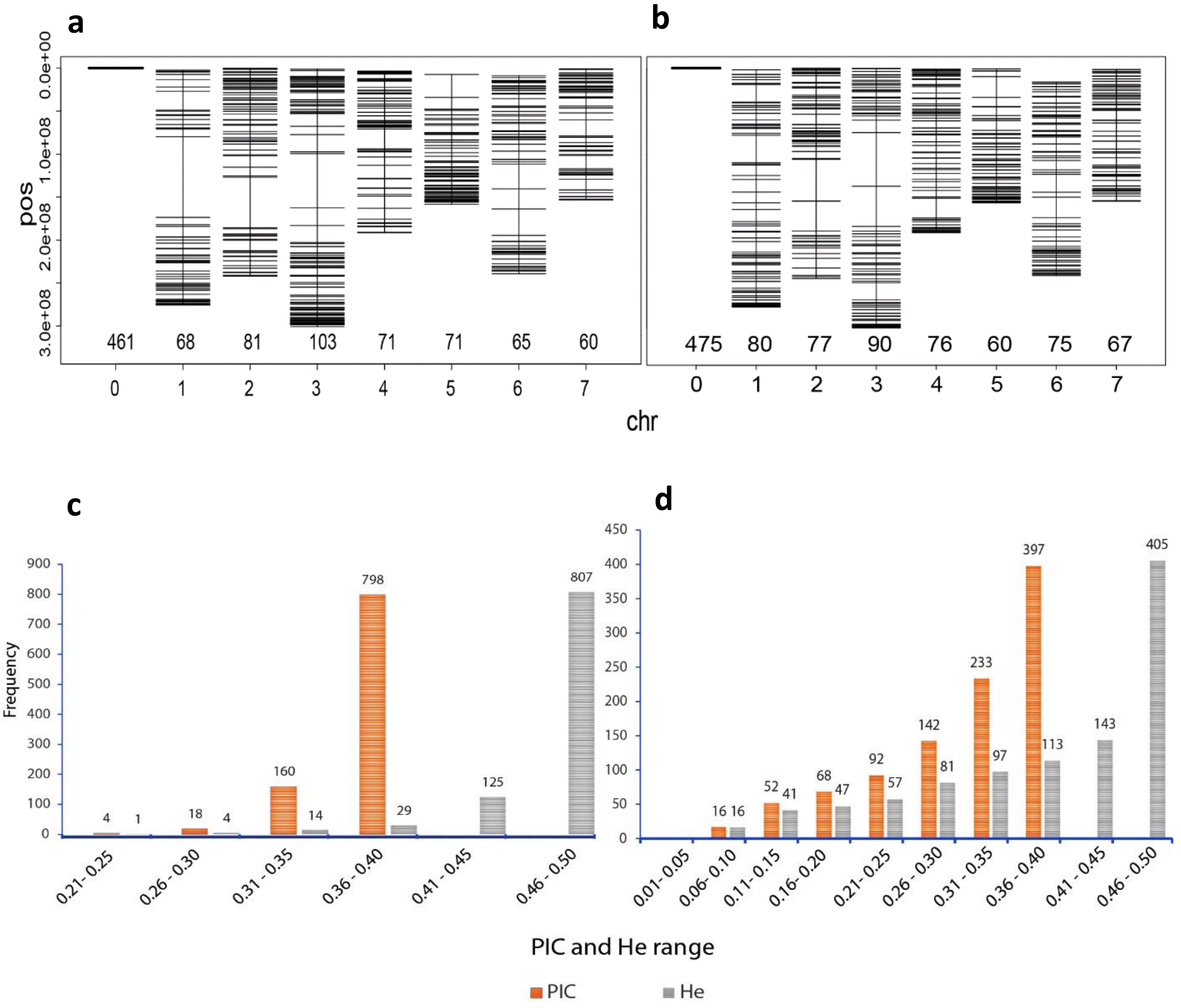

Supplementary Figure S2. Distribution of 980 SNPs (a) and 1000 silicoDArTs (b) across the pearl millet genome. The markers that were not mapped indicated by 0. These markers selected for diversity analysis. In (c) and (d), the distribution of polymorphic information content (PIC) of SilicoDArTs (orange) and SNPs (grey) are shown. More than 98 % of the SNP markers had PIC and He values of above 0.3.

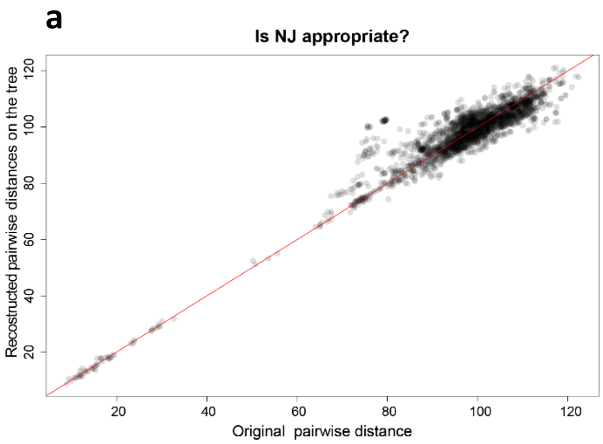

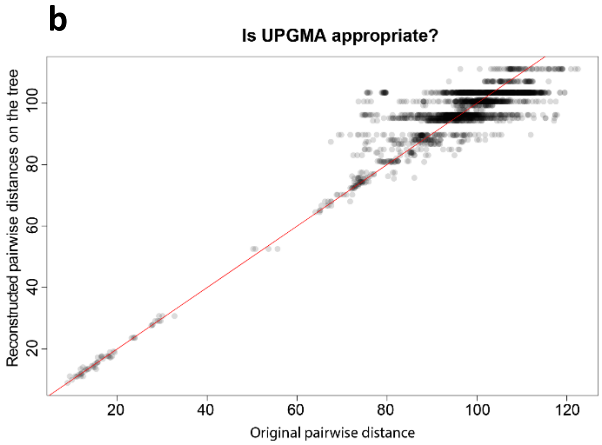

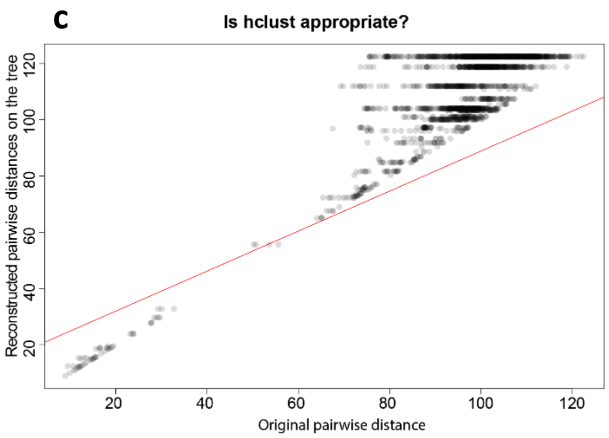

Supplementary Figure S3. Plots of the original distance matrix against the recalculated distance during tree inference for NJ (a), UPGMA (b), and hclust (c). In hclust, the linear regression line shows deviation, indicating that in hierarchical clustering the recalculated distance during tree inference was less represent the original distance matrix. Hence, hierarchical clustering is less suitable to show the diversity of the population in this specific data type

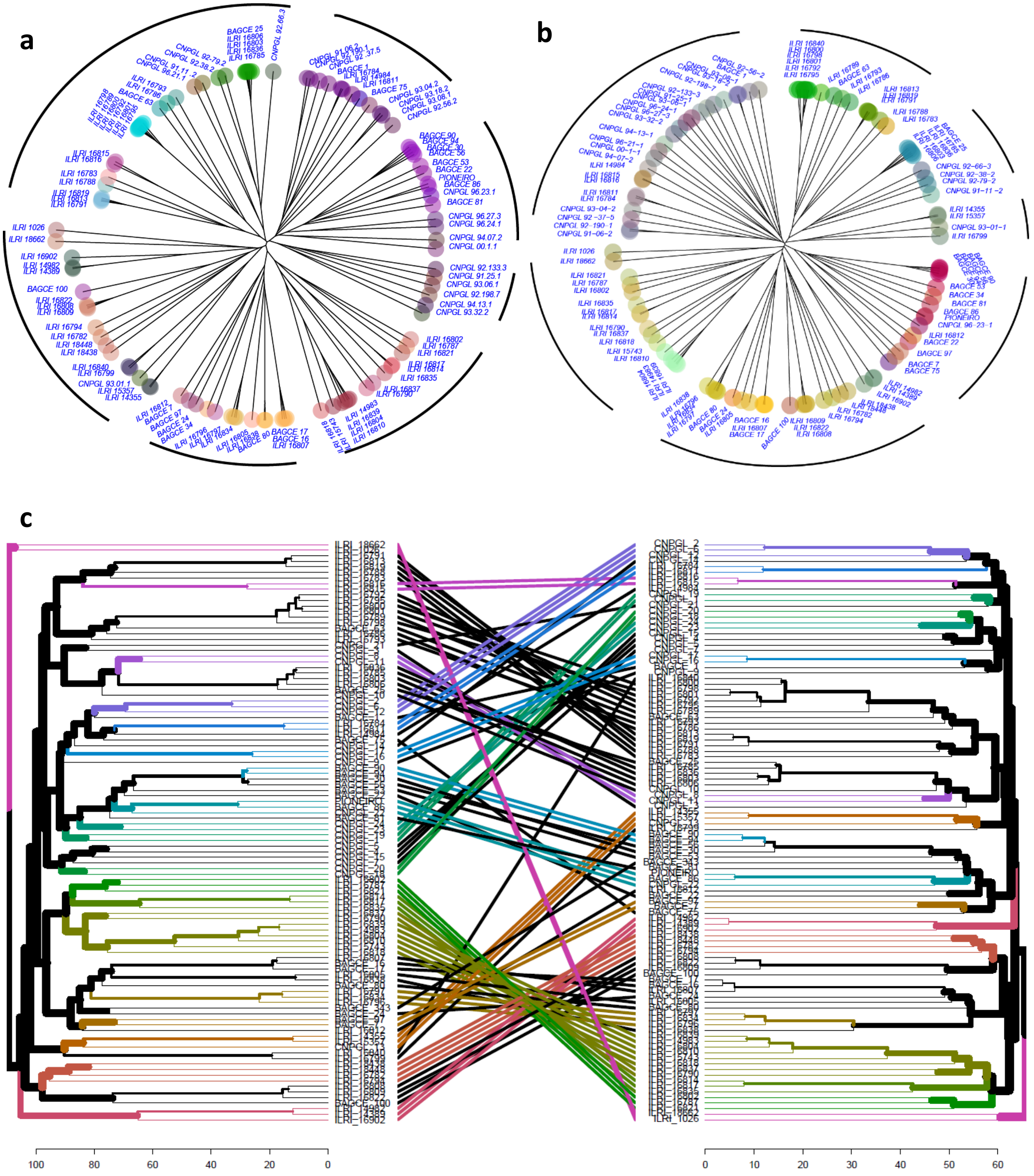

Supplementary Figure S4. Comparisons in clustering between the 980 SNPs and 1000 SilicoDArTs selected for diversity analysis. UPGMA using the 980 SNPs (a), the 1000 SilicoDArTs (b), A tanglegram comparing the two-clustering produced by the data sets (c). Cophenetic correlation and correlation analysis using the dendextend r-package (Galili 2015) showed 0.86 correlation coefficient between the dendrograms produced by the 980 SNPs and 1000 SilicoDArTs.

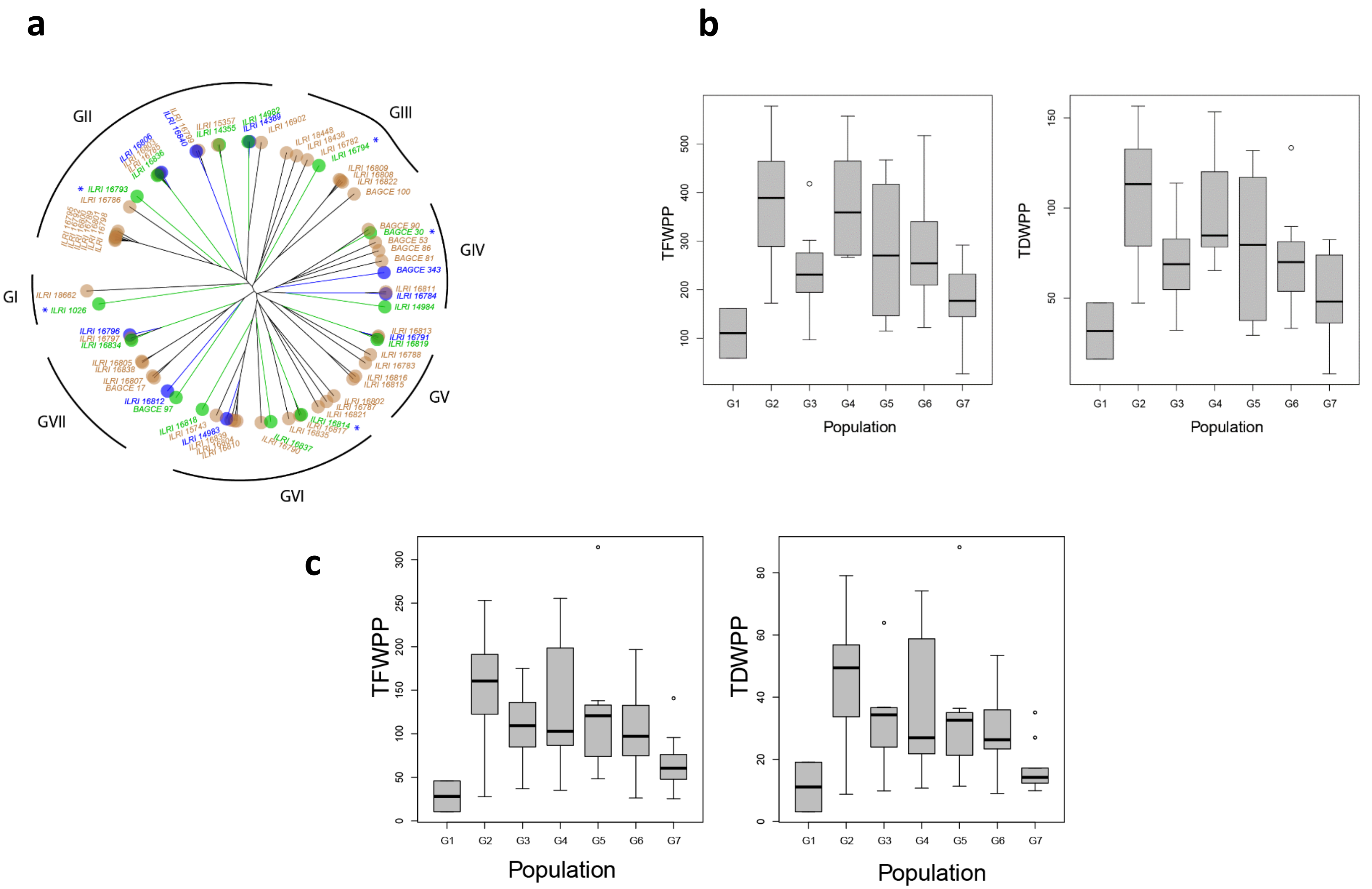

Supplementary Figure S5. Comparison of mean phenotypic variability among groups (GI to GVII). (a), UPGMA tree for 68 accessions used in subsetting, showing seven groups (b), box plots showing mean of total fresh-weight per plant (TFWPP) and total dry-weight (TDWPP) for each group under optimum water conditions (c), under water deficit conditions.
